# Mind-body therapy for treating fibromyalgia: a systematic review

**DOI:** 10.1093/pm/pnae076

**Published:** 2024-08-02

**Authors:** Jeremy P Steen, Vivek Kannan, Abdullah Zaidi, Holger Cramer, Jeremy Y Ng

**Affiliations:** Institute of Health Policy, Management, and Evaluation, Dalla Lana School of Public Health, University of Toronto, Toronto, ON M5T 3M6, Canada; Institute for General Practice and Interprofessional Care, University Hospital Tübingen, Tübingen 72076, Germany; Robert Bosch Center for Integrative Medicine and Health, Bosch Health Campus, Stuttgart 70376, Germany; Institute for General Practice and Interprofessional Care, University Hospital Tübingen, Tübingen 72076, Germany; Robert Bosch Center for Integrative Medicine and Health, Bosch Health Campus, Stuttgart 70376, Germany; Institute for General Practice and Interprofessional Care, University Hospital Tübingen, Tübingen 72076, Germany; Robert Bosch Center for Integrative Medicine and Health, Bosch Health Campus, Stuttgart 70376, Germany; Institute for General Practice and Interprofessional Care, University Hospital Tübingen, Tübingen 72076, Germany; Robert Bosch Center for Integrative Medicine and Health, Bosch Health Campus, Stuttgart 70376, Germany; Institute for General Practice and Interprofessional Care, University Hospital Tübingen, Tübingen 72076, Germany; Robert Bosch Center for Integrative Medicine and Health, Bosch Health Campus, Stuttgart 70376, Germany; Centre for Journalology, Ottawa Methods Centre, Ottawa Hospital Research Institute, Ottawa, ON K1H 8L6, Canada

**Keywords:** Fibromyalgia, chronic pain, mind-body therapy, mind-body interventions, meditation, systematic review

## Abstract

**Objective:**

Fibromyalgia (FM) is a chronic and disabling condition that presents treatment challenges for both patients and healthcare providers. The objective of this review was to systematically assess the effectiveness and safety of mind-body therapies for FM.

**Methods:**

We searched MEDLINE, EMBASE, PsycINFO, AMED, and CINAHL databases from their inception to December 2023. Eligible articles included adults diagnosed with FM participating in a mind-body therapy intervention and were published from the beginning of 2012 onwards. We assessed the quality of the studies using the Joanna Briggs Institute Critical Appraisal Checklists.

**Results:**

Twenty-seven studies (1969 participants) were included, comprising 22 randomized controlled trials and 5 quasi-experimental studies. Mind-body therapies included guided imagery (n = 5), mindfulness-based stress reduction (n = 5), qi gong (n = 5), tai chi (n = 5), biofeedback (n = 3), yoga (n = 2), mindfulness awareness training (n = 1), and progressive muscle relaxation (n = 1). With the exception of mindfulness-based stress reduction, all therapies had at least 1 study showing significant improvements in pain at the end of treatment. Three or more studies on qi gong and tai chi demonstrated significant improvements in fatigue and multidimensional function, with tai chi showing the most evidence for improvement in anxiety and depression. Approximately one-third of the studies reported on adverse events.

**Conclusions:**

This systematic review found that mind-body therapies are potentially beneficial for adults with FM. Further research is necessary to determine if the positive effects observed post-intervention are sustained.

**Study registration:**

Open Science Framework (https://osf.io) (September 12, 2023; https://doi.org/10.17605/osf.io/6w7ac).

## Introduction

Fibromyalgia (FM) is a complex, chronic condition, characterized by generalized pain, functional impairment, fatigue, and sleep disturbances.^1^ Its estimated prevalence ranges from 0.2% to 6.6% in the general population,^2^ with a notable predominance in females and higher rates observed in specific risk groups.^3^ According to the American College of Rheumatology, FM is diagnosed in adults when the following criteria are met: generalized pain, defined as pain in at least 4 of 5 regions; symptoms that have persisted at a similar level for at least 3 months; and significant symptom severity.^4^ Conventional treatments often include medications such as gabapentin and pregabalin,^5^^,^^6^ which are also used in epilepsy management; however, these treatments are frequently associated with adverse events (AEs), and many patients continue to experience disability.^7^^,^^8^ Recent studies suggest that managing FM should involve a comprehensive approach integrating both pharmacological and non-pharmacological therapies.^9^

Mind-body therapies are broadly defined as a group of therapies that target “interactions among the brain, mind, body, and behavior, with the intent to use the mind to affect physical functioning and promote health.”^10^ Examples include mindfulness (which foster conscious and present-centered awareness and a non-judgmental perspective, such as mindfulness-based stress reduction [MBSR] and meditation awareness training [MAT]);^11^ biofeedback (which uses technology to provide auditory or visual feedback on physiological processes, such as breathing, heart rate, or brain waves, helping individuals gain greater control over their bodily functions);^12^ movement therapies (such as yoga, tai chi, and qi gong, which enhance the harmony and circulation of the body’s subtle energy system and increase joint flexibility, muscle strength, and balance)^13^^,^^14^; and relaxation therapy (such as autogenic training, guided imagery, and progressive muscle relaxation [PMR], which aim to induce the body’s relaxation response).^15^

Although several reviews have been published on the effects of mind-body therapies for treating FM,^16–18^ they have certain limitations. Two of the reviews did not formally assess the methodological quality of the included studies,^16^^,^^17^ which is critical for contextualizing findings and generating recommendations.^19^^,^^20^ The third review^18^ is outdated due to the rapid expansion of research in this field in recent years, with many new trials and primary studies now available. As a result, the body of evidence has evolved considerably, highlighting the need for a fresh review that incorporates the latest findings to ensure recommendations are current and relevant to the evolving landscape of FM treatment and management through mind-body therapies.

Our systematic review aimed to (1) summarize the existing evidence on the effectiveness and safety of mind-body therapies for adults with FM and (2) identify gaps in the published evidence to guide potential avenues for future intervention work. We improved upon the limitations of previous reviews by addressing a comprehensive range of patient-important outcomes, including pain, fatigue, patient global impression of change (PGIC), multidimensional function, sleep disturbance, depression, anxiety, and AEs. Additionally, our review used an approved method to evaluate the methodological quality of eligible studies, resulting in a more up-to-date and comprehensive overview of the field.^21^

## Methods

We conducted this systematic review using the Joanna Briggs Institute (JBI) systematic review process.^21–23^ We followed the Preferred Reporting Items for Systematic Reviews and Meta-Analyses (PRISMA 2020) guidelines^24^ and preregistered our review on the Open Science Framework (registration DOI: 10.17605/osf.io/6w7ac) prior to conducting the search. Initially, we planned to perform a scoping review on the use of mind-body therapies for treating FM. However, after conducting preliminary literature searches and assessing our objectives, we determined that a systematic review would be more appropriate for 2 main reasons.^25^ First, our goal was to inform clinical decision-making by assessing the effectiveness and safety of mind-body therapies for FM, which is best achieved through a systematic review. Second, because the field of mind-body therapy for FM is well-established, with a considerable number of existing primary studies, a systematic review provides a more appropriate methodological framework for updating and synthesizing the available evidence.

### Eligibility criteria

#### Participants

The target population was adults (≥18 years of age) with a clinical diagnosis of FM, as defined by any recognized diagnostic criteria.^4^^,^^26–28^

#### Intervention

We included interventions that incorporated at least one type of mind-body therapy. Based on criteria from the National Center for Complementary and Integrative Health (NCCIH),^29^ we *a priori* decided to include the following types of mind-body therapy: autogenic training, biofeedback, MBSR, MAT, guided imagery, PMR, tai chi, qi gong, and yoga. We excluded interventions delivered manually by a therapist to a participant (such as massage, acupuncture, and physiotherapy) since these do not actively engage participants in their treatment, which is a key component of mind-body interventions according to the NCCIH definition.^29^ Studies on psychological therapies were excluded to avoid overlap with several systematic reviews that focus on specific forms of psychological therapy for FM (eg, group psychotherapy and cognitive-behavioral therapy [CBT]).^30–35^ We also excluded interventions involving spiritual prayer, art or dance therapy, as well as patient educational programs (eg, FM self-management programs) since these are generally accepted within the biomedical system of care.^36–38^

#### Outcomes of interest

To inform our choice of outcomes, we consulted the Initiative on Outcome Measures in Rheumatology Clinical Trials (OMERACT) core outcome set.^39^ We then assessed the frequency of reporting across eligible studies to determine the most important outcomes for patients, in line with Grading of Recommendations Assessment, Development and Evaluation (GRADE) guidelines.^40^ The outcomes of interest included: (1) pain, (2) fatigue, (3) PGIC,^41^ (4) multidimensional function, (5) sleep disturbance, (6) depression, (7) anxiety, and (8) AEs. Since we anticipated that eligible studies would also report on other health-related outcomes (eg, perceived stress, self-efficacy, cognitive function, pain catastrophizing, pressure pain threshold, and tender points), we presented data on other health-related outcomes in a seperate table (Table 5).

#### Types of studies

We included primary studies published in the past 12 years (from 2012 through December 2023) to avoid overlap with a previous systematic review on CBT and mind-body therapies for FM,^18^ which included studies up to October 2013. We limited our review to studies published in peer-reviewed journals and available in English. We excluded editorials, commentaries, case reports, case series, abstracts, letters, and protocols.

### Search strategy and information sources

Following the JBI 3-step search strategy,^23^ an initial search was conducted across MEDLINE and EMBASE to identify keywords and index terms from titles and abstracts. We consulted an experienced medical researcher (J.Y.N.) specializing in knowledge synthesis to ensure a comprehensive list of terms. All identified keywords and indexing terms were then used to tailor searches for each academic database, including MEDLINE, EMBASE, PsycINFO, AMED, and CINAHL. We also searched the reference lists of existing scoping and systematic reviews on the topic to identify any additional relevant studies. The literature was searched from inception until December 29, 2023. Our full search strategy is available at https://osf.io/quxtc.

### Study selection

Three reviewers (J.P.S., V.K., and A.Z.) screened titles and abstracts for initial eligibility and reviewed the full text of potentially eligible studies, independently and in duplicate. Disagreements were resolved through discussion or third-party adjudication as needed.

### Data extraction and quality assessment

Using standardized, piloted forms, 3 reviewers (J.P.S., V.K., and A.Z.) conducted calibration exercises and independently extracted data on study design, objectives, FM definition, participant characteristics, interventions, comparators (if present), and outcomes of interest. Disagreements were resolved by discussion or third-party adjudication if necessary. The primary assessment of outcomes occurred post-intervention, directly following the end of treatment. This time point was chosen to maximize the likelihood of detecting treatment effects, as effects were expected to be most pronounced immediately after the mind-body therapy intervention. Outcomes were also assessed at follow-up (ie, after the end of the treatment period) when available.

Three reviewers (J.P.S., V.K., and A.Z.), independently and in duplicate, assessed the methodological quality of eligible studies using the JBI Critical Appraisal Checklist for randomized controlled trials (RCTs) and quasi-experimental studies.^21^ The checklist consists of 13 domains for RCTs and 9 domains for quasi-experimental studies, with each domain rated as yes, no, unclear, or not applicable. Disagreements were resolved by discussion or third-party adjudication as needed. The results of the quality assessments were reported in tables and summarized narratively.

### Data synthesis and analysis

The main findings were summarized and presented using tables and qualitative analysis of descriptive data. For the qualitative analysis, 3 reviewers (J.P.S., V.K., and A.Z.) identified codes based on the main topics discussed in the articles and organized them into thematic groups. The reviewers then created a narrative to discuss how the findings related to the research question and identified knowledge gaps in the existing literature.

## Results

### Search results

**Figure 1.**
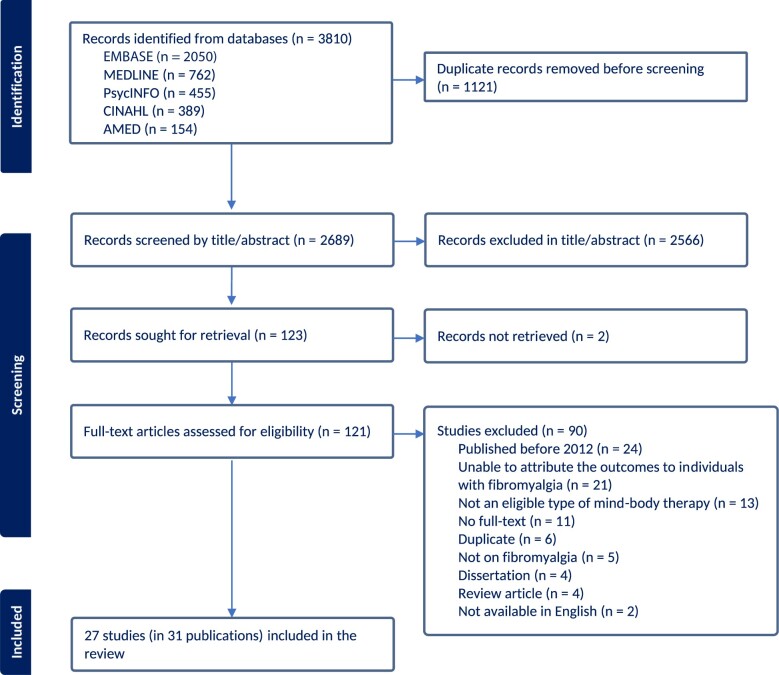
PRISMA flow diagram. AMED, Allied and Complementary Medicine; APA, American Psychological Association; CINAHL, Cumulative Index to Nursing and Allied Health Literature; EMBASE, Excerpta Medica Database.

Searches retrieved 2689 items following deduplication, of which 2566 titles and abstracts were eliminated, leaving 121 full-text articles to be considered. Of those, 90 were considered ineligible for various reasons. This left 27 studies (31 publications) for inclusion in this systematic review.^42–72^ In Figure 1, a PRISMA diagram can be found depicting this process.

### Characteristics of Eligible Studies

Twenty-seven studies meeting the inclusion criteria were published between 2012 and 2022, spanning across 10 countries: Spain (n = 9),^42–44^^,^^55^^,^^57–61^^,^^65^^,^^67^ the United States (n = 7),^46^^,^^47^^,^^49^^,^^51^^,^^52^^,^^56^^,^^62^ Canada (n = 2),^53^^,^^63^^,^^64^ Germany (n = 2),^45^^,^^48^^,^^66^ South Korea (n = 2),^70^^,^^72^ England (n = 1),^68^ Israel (n = 1),^50^ Italy (n = 1),^54^ Taiwan (n = 1),^71^ and the Netherlands (n = 1).^69^ Of the 27 studies (comprising 1969 participants), 5 were quasi-experimental studies,^44^^,^^51^^,^^61^^,^^63^^,^^65^ while the remaining 22 were RCTs. The RCTs included various comparison groups: attention control interventions (such as sham or peer group support) (n = 10), usual care (n = 7), and wait-list conditions (n = 5). The eligible studies encompassed a range of mind-body therapies, including 5 studies on guided imagery,^50^^,^^56–58^^,^^69^^,^^71^ 5 on MBSR,^43^^,^^47^^,^^48^^,^^55^^,^^59^^,^^66^ 5 on qi gong,^52^^,^^53^^,^^60^^,^^62–64^ 5 on tai chi,^49^^,^^54^^,^^61^^,^^65^^,^^70^ 3 on biofeedback,^42^^,^^44^^,^^45^^,^^67^ 2 on yoga,^46^^,^^51^ 1 on MAT,^68^ and 1 on PMR.^72^

The duration of treatments in these studies ranged from 2 weeks to 12 months, with an average length of 12 weeks. More than half of the studies (n = 7) included only female participants.^42–48^^,^^50–52^^,^^55^^,^^56^^,^^60–62^^,^^64^^,^^66^^,^^67^^,^^70^ Participants were recruited from diverse settings, including primary care clinics (n = 7),^42^^,^^44^^,^^45^^,^^50^^,^^51^^,^^59^^,^^67^^,^^71^ FM self-help and/or support groups (n = 5),^57^^,^^58^^,^^60^^,^^61^^,^^65^^,^^69^ hospital electronic medical records (n = 3),^43^^,^^55^^,^^62^ and a tertiary care center (n = 1).^46^ Four studies recruited participants from both primary care clinics and FM self-help/support groups,^48^^,^^49^^,^^52^^,^^66^^,^^68^ while in 7 studies, the recruitment setting was not clearly specified.^47^^,^^53^^,^^54^^,^^56^^,^^63^^,^^64^^,^^70^^,^^72^  Table 1 provides a summary of the general characteristics of the eligible studies, including the first author, year of publication, study design, country, recruitment setting, sample size, age, and definition of FM.

**Table 1.** General characteristics of the included studies.

Author and year	Title	Study design	Country of participant population	Ethnicity of participant population	Where FM patients were recruited from	Sample size	Mean age (standard deviation)	Proportion female (%)	Definition of FM
Alba 2022^42^; Terrasa 2020^67^	EEG-heart rate connectivity changes after sensorimotor rhythm neurofeedback training: ancillary study.	Parallel RCT	Spain	Spanish	Primary care	n = 17	54.94 (10.11)	100	No definition provided.
Andres-Rodriguez 2019^43^	Immune-inflammatory pathways and clinical changes in fibromyalgia patients treated with mindfulness-based stress reduction (MBSR): a randomized, controlled clinical trial.	Parallel RCT	Spain	Spanish	In-patient hospital database	n = 70	53.30 (8.06)	100	FM is a disabling syndrome characterized by chronic widespread musculoskeletal pain, increased pain sensitivity including allodynia and hyperalgesia with tenderness to touch but no known structural pathology in muscles, ligaments, or joints.
Barbosa-Torres 2021^44^	Clinical findings in SMR neurofeedback protocol training in women with fibromyalgia syndrome.	Quasi-experimental with no control	Spain	Spanish	Primary care	n = 37	54.92 (7.89)	100	FM is a condition generally associated with multiple symptoms, such as sleep deprivation, tiredness, chronic fatigue, and cognitive impairment. Its predominant characteristic is non-articular, widespread, chronic muscular-skeletal pain in specific areas all over the body. The psychological factors most frequently found in FM patients are intense negative emotions (anxiety, depression), a maladaptive coping style, an unadjusted attention pattern, and an excessive worry response.
Baumueller 2017^45^	Electromyogram biofeedback in patients with fibromyalgia: a randomized controlled trial.	Parallel RCT	Germany	German	Primary care	n = 40	55.70 (6.02)	100	FM is a chronic pain syndrome with a high burden for the individual person and for society. FM is characterized by chronic widespread pain, sleep disturbances, and additional symptoms such as fatigue and depression. Some patients report a decline in memory, cognitive function, and mental alertness. Activities of daily living, working ability, and quality of life are considerably limited.
Carson 2012^46^	Follow-up of yoga of awareness for fibromyalgia: results at 3 months and replication in the wait-list group.	Parallel RCT	United States	White; Native American; Other	University tertiary care center	n = 53	55.40 (11.30)	100	No definition provided.
Cash 2015^47^	Mindfulness meditation alleviates fibromyalgia symptoms in women: results of a randomized clinical trial.	Parallel RCT	United States	European	News media	n = 91	NR	100	According to the American College of Rheumatology guidelines, a patient satisfies diagnostic criteria for FM if they report widespread pain and high symptom severity that have persisted for at least 3 months with no alternate explanation. Fatigue is one of the most prevalent symptoms reported by women with FM and may have the greatest impact of any symptom experienced.
Grossman 2017^48^; Schmidt 2011^66^	Mindfulness-based intervention does not influence cardiac autonomic control or the pattern of physical activity in fibromyalgia during daily life.	Parallel RCT	Germany	White; Other	Primary care, in-patient hospital database, news media, and patient self-help groups	n = 168	54.00 (8.55)	100	FM is a clinical functional disorder with major symptoms of chronic widespread pain, fatigue, stiffness, and sleep disturbance.
Jones 2012^49^	A randomized controlled trial of 8-form tai chi improves symptoms and functional mobility in fibromyalgia patients.	Parallel RCT	United States	White; Other	Primary care, news media, FM support group, and Oregon Pain Society	n = 101	54.00 (NR)	92.82	FM is a common, multisymptomatic chronic pain illness with significant functional mobility limitations. People with FM suffer from widespread musculoskeletal pain, fatigue, stiffness, disturbed sleep, and declining physical function.
Kaplun 2021^50^	Effects of brief guided imagery on female patients diagnosed with fibromyalgia: an exploratory controlled trial.	Parallel RCT	Israel	Israeli; Russian; European/American; Asian/African	Primary care	n = 37	58.70 (8.80)	100	FM is a syndrome characterized by chronic pain and moderate or severe fatigue accompanied by a lack of energy and exhaustion.
Lazaridou 2019^51^	Impact of daily yoga-based exercise on pain, catastrophizing, and sleep amongst individuals with fibromyalgia.	Quasi-experimental with no control	United States	Caucasian; African American	Primary care and news media	n = 46	48.50 (13.90)	100	FM is a chronic widespread pain disorder characterized by negative affect, sleep disturbance, and fatigue.
Liu 2012^52^	Benefit of qigong exercise in patients with fibromyalgia: a pilot study.	Parallel RCT	United States	American	Primary care and FM support group	n = 14	56.65 (NR)	100	FM is a disabling disorder featuring widespread chronic pain and other often debilitating symptoms including fatigue, sleep difficulties, and depressed mood that contribute to a high level of functional disability.
Lynch 2012^53^; Sawynok 2014^64^	Lynch 2012: A randomized controlled trial of qigong for fibromyalgia. Sawynok 2014: Qualitative analysis of a controlled trial of qigong for fibromyalgia: advancing understanding of an emerging health practice.	Parallel RCT	Canada	Canadian	Lynch 2012: News media Sawynok 2014: NR	Lynch 2012: n = 100 Sawynok 2014: n = 73	Lynch 2012: 52.00 (8.75) Sawynok 2014: 52.00 (original RCT); 53.00 (extension trial) (NR)	Lynch 2012: 96.00 Sawynok 2014: 73.00	Lynch 2012: FM is a chronic pain condition characterized by widespread musculoskeletal pain of greater than 3 months duration, and it is associated with fatigue and sleep disturbance as well as other symptoms. Sawynok 2014: A chronic pain condition associated with sleep disturbances, fatigue, and decreased quality of life.
Maddali-Bongi 2016^54^	Efficacy of rehabilitation with Tai Ji Quan in an Italian cohort of patients with fibromyalgia syndrome.	Parallel RCT	Italy	Italian	NR	n = 44	52.24 (12.19)	NR	FM syndrome is a rheumatic disease, characterized by chronic widespread pain for more than 3 months and other symptoms such as fatigue, non-restorative sleep, and cognitive and somatic symptoms (headache, depressive symptoms, and irritable bowel).
Medina 2022^55^	Differential brain perfusion changes following two mind-body interventions for fibromyalgia patients: an arterial spin labelling fMRI study.	Parallel RCT	Spain	Spanish	In-patient hospital database	n = 90	52.61 (8.36)	100	FM syndrome is a chronic condition characterized by widespread musculoskeletal pain, fatigue, stiffness, sleep disturbances, cognitive problems, and distress.
Menzies 2014^56^	Effects of guided imagery on biobehavioral factors in women with fibromyalgia.	Parallel RCT	United States	Hispanic/Latino; Other	News media	n = 72	46.90 (12.80)	100	FM is characterized by widespread pain and tenderness on examination, accompanied by somatic symptoms such as fatigue, and psychological symptoms such as depression.
Onieva-Zafra 2015^57^, 2019^58^	Onieva-Zafra 2015: Effectiveness of guided imagery relaxation on levels of pain and depression in patients diagnosed with fibromyalgia. Onieva-Zafra 2019: Benefits of a home treatment program using guided imagery relaxation based on audio recordings for people with fibromyalgia.	Parallel RCT	Spain	Spanish	Various FM associations	n = 60	52.47 (6.17)	Onieva-Zafra 2015: 3.60 Onieva-Zafra 2019: 96.40	Onieva-Zafra 2015: FM is a chronic disease that affects the life of the patient on all levels (social, personal, and professional), and thus, an interdisciplinary approach to its treatment is required. Onieva-Zafra 2019: FM is a frequently diagnosed pain disorder primarily affecting women. It has a high comorbidity often accompanied by symptoms such as morning stiffness, fatigue, anxiety, depression, insomnia, and reduced cognitive performance.
Perez-Aranda 2019^59^	A randomized controlled efficacy trial of mindfulness-based stress reduction compared with an active control group and usual care for fibromyalgia: the EUDAIMON study.	Parallel RCT	Spain	Spanish	Primary care	n = 225	53.27 (7.93)	98.22	FM is mainly characterized by chronic widespread pain, fatigue, stiffness, sleep problems, perceived cognitive dysfunction, and distress.
Rodriguez-Mansilla 2021^60^	Effects of non-pharmacological treatment on pain, flexibility, balance and quality of life in women with fibromyalgia: a randomised clinical trial.	Parallel RCT	Spain	Spanish	Various FM associations	n = 141	52.24 (6.19)	100	FM is diffuse and widespread pain in combination with the presence of multiple tender points. In addition to pain, these patients have sensory symptoms, such as paraesthesia, motor symptoms, such as muscle stiffness, contractures and tremors, and vegetative symptoms, such as tingling sensations.
Romero-Zurita 2012^61^	Effectiveness of a tai-chi training and detraining on functional capacity, symptomatology and psychological outcomes in women with fibromyalgia.	Quasi-experimental with no control	Spain	Spanish	Various local FM associations	n = 32	51.35 (6.75)	100	FM is a chronic diffuse pain condition that probably results from abnormal central pain processing. The symptoms most frequently are chronic pain, characterized by generalized pain, stiffness, fatigue, disturbed sleep, psychological distress, and impaired cognitive function.
Sarmento 2020^62^	The therapeutic efficacy of qigong exercise on the main symptoms of fibromyalgia: a pilot randomized clinical trial.	Parallel RCT	United States	American	Primary care, and emergency or urgent care hospital database	n = 28	49.40 (13.19)	100	FM is a chronic syndrome that was recognized as a clinical entity in 1990. The most prevalent and debilitating symptoms of FM are widespread chronic pain, chronic fatigue, and sleep disturbances.
Sawynok 2013^63^	Extension trial of qigong for fibromyalgia: a quantitative and qualitative study.	Quasi-experimental with no control	Canada	Canadian	Previous participants invited to participate in the extension trial	n = 20	53.00 (9.30)	100	FM is a chronic pain condition associated with sleep and mood disturbances, and diminished quality of life.
Segura-Jimenez 2014^65^	Effectiveness of tai-chi for decreasing acute pain in fibromyalgia patients.	Quasi-experimental with no control	Spain	Spanish	Various FM associations	n = 43	51.70 (6.40)	96.43	No definition provided.
Van-Gordon 2017^68^	Meditation awareness training for the treatment of fibromyalgia syndrome: a randomized controlled trial.	Parallel RCT	England	British; White (Non-British); Asian; Black (Caribbean)	Primary care, news media, and various FM associations/support groups	n = 148	46.90 (9.43)	83.10	FM syndrome is a chronic pain disorder that affects approximately 3% of adults, with higher rates of occurrence in females compared to males. Individuals with FM syndrome typically experience symptoms of widespread musculoskeletal pain, sleep disturbance, poor quality of life, cognitive dysfunction (particularly memory impairment), psychological distress (eg, depression, anxiety, and stress), and fatigue.
Verkaik 2014^69^	Guided imagery in people with fibromyalgia: a randomized controlled trial of effects on pain, functional status and self-efficacy.	Parallel RCT	Netherlands	Dutch	News media and various FM associations	n = 65	47.50 (11.38)	98.46	FM is a complex rheumatic disorder that affects up to 5% of the general population worldwide. In addition to chronic widespread pain, patients often experience fatigue, disturbed sleep, stiffness, reduced functioning, and cognitive problems.
Wong 2018^70^	Effectiveness of tai chi on cardiac autonomic function and symptomatology in women with fibromyalgia: a randomized controlled trial.	Parallel RCT	Korea	Korean	NR	n = 37	51.00 (1.97)	100	FM is an idiopathic disease affecting approximately 3% of the world population, primarily diagnosed in middle-aged women. Although FM is mainly characterized by chronic pain and fatigue, reduced muscular strength and flexibility are common symptoms associated with the presentation of the disorder.
Wu 2021^71^	Effects of neurofeedback on fibromyalgia: a randomized controlled trial.	Parallel RCT	Taiwan	Taiwanese	Primary care	n = 80	47.00 (13.13)	88.75	FM is a condition characterized by widespread pain, memory problems, sleep disturbances, and cognitive impairment. FM frequently co-occurs with irritable bowel syndrome, fatigue, depression, anxiety disorders, and poor quality of life.
Yoo 2022^72^	Effects of progressive muscle relaxation therapy with home exercise on pain, fatigue, and stress in subjects with fibromyalgia syndrome: a pilot randomized controlled trial.	Parallel RCT	Korea	Korean	NR	n = 37	NR (Range: 20-65)	89.19	FM is a chronic widespread pain disorder of the musculoskeletal system and is accompanied by symptoms of fatigue, depression, sleep disorders, and physical and mental stress.

Abbreviations: FM, fibromyalgia; NR, not reported; RCT, randomized controlled trial.

### Quality assessment

Among the 22 RCTs, the methodological quality scores ranged from 5 to 11 (mean = 7.9; SD = 1.6) out of a maximum of 13 points (Table 2). Eighteen of the 22 RCTs used true randomization, while in the remaining 4 trials, the process of randomization was not clearly described.^46^^,^^52^^,^^54^^,^^57^^,^^58^ Concealment of group allocation occurred in approximately one-third of the RCTs^45^^,^^48^^,^^53^^,^^60^^,^^62^^,^^64^^,^^68^^,^^69^^,^^71^ and was unclear in the remaining two-thirds. In many of the included RCTs, blinding did not occur due to practical difficulties. Among the 5 quasi-experimental studies, the methodological quality scores ranged from 6 to 7 (mean = 6.8; SD = 0.4) out of a maximum of 9 points (Table 3).

**Table 2.** Quality assessment of the included RCTs.

Author and year	Q1	Q2	Q3	Q4	Q5	Q6	Q7	Q8	Q9	Q10	Q11	Q12	Q13	Score/13
Alba 2022^42^	Y	Unclear	N	Unclear	N	Y	Unclear	Y	Y	Y	Unclear	N	Y	6/13
Andres-Rodriguez 2019^43^	Y	Unclear	Y	Unclear	N	Y	Y	Y	Y	N	Y	N	Y	8/13
Baumueller 2017^45^	Y	Y	N	N	N	Y	Y	Y	Y	Y	Unclear	N	Y	8/13
Carson 2012^46^	Unclear	Unclear	Y	Unclear	N	Unclear	Y	Y	Y	Y	Y	N	Y	7/13
Cash 2015^47^	Y	Unclear	N	Unclear	N	Unclear	Unclear	Y	Y	Y	Y	N	Y	6/13
Grossman 2017^48^	Y	Y	Unclear	Y	N	Unclear	Unclear	Unclear (Y for cardiorespiratory function and ambulatory accelerometry outcomes)	Y	Y	Y	Y	Y	8/13
Jones 2012^49^	Y	Unclear	Y	Unclear	N	Y	Y	Y	Y	Y	Y	N	Y	9/13
Kaplun 2021^50^	Y	Unclear	Y	Unclear	N	Unclear	Unclear	Y	Y	Y	Unclear	N	Y	6/13
Liu 2012^52^	Unclear	Unclear	N	Unclear	N	Y	Y	Y	Y	Y	Unclear	N	Y	6/13
Lynch 2012^53^; Sawynok 2014^64^	Y	Y	N	N	N	N	Y	Y	Y	Y	Unclear	N	Y	7/13
Maddali-Bongi 2016^54^	Unclear	Unclear	Y	Unclear	N	Unclear	Unclear	Y	Y	Y	Unclear	N	Y	5/13
Medina 2022^55^	Y	Unclear	Y	Y	N	Y	Unclear	Y	Y	N	Y	Y	Y	9/13
Menzies 2014^56^	Y	Unclear	Y	Unclear	N	Y	Unclear	Y	Y	Y	Y	Y	Y	9/13
Onieva-Zafra 2015^57^, 2019^58^	Unclear	Unclear	Y	N	N	N	N	Y	Y	Y	Unclear	N	Y	5/13
Perez-Aranda 2019^59^	Y	Unclear	Y	Unclear	N	Unclear	Unclear	Y	Y	Y	Y	Y	Y	8/13
Rodriguez-Mansilla 2021^60^	Y	Y	N	N	N	Y	Y	Y	Y	Y	Unclear	Y	Y	9/13
Sarmento 2020^62^	Y	Y	N	Y	N	Y	Y	Y	Y	Y	Unclear	N	Y	9/13
Van Gordon 2017^68^	Y	Y	Y	Y	N	Y	Y	Y	Y	Y	Y	N	Y	11/13
Verkaik 2014^69^	Y	Y	Y	N	N	Y	Unclear	Y	Y	Y	Y	Y	Y	10/13
Wong 2018^70^	Y	Unclear	Y	Unclear	N	Y	Y	Y	Y	Y	Unclear	Y	Y	9/13
Wu 2021^71^	Y	Y	Y	N	N	Y	Y	Y	Y	N	Y	Y	Y	10/13
Yoo 2022^72^	Y	Unclear	Y	N	N	Unclear	Y	Y	Y	Y	Unclear	Y	Y	8/13

Abbreviations: Y, yes; N, no.

1. Was true randomization used for assignment of participants to treatment groups?

2. Was allocation to treatment groups concealed?

3. Were treatment groups similar at the baseline?

4. Were participants blind to treatment assignment?

5. Were those delivering the treatment blind to treatment assignment?

6. Were treatment groups treated identically other than the intervention of interest?

7. Were outcomes assessors blind to treatment assignment?

8. Were outcomes measured in the same way for treatment groups?

9. Were outcomes measured in a reliable way?

10. Was follow-up complete and if not, were differences between groups in terms of their follow-up adequately described and analyzed?

11. Were participants analyzed in the groups to which they were randomized?

12. Was appropriate statistical analysis used?

13. Was the trial design appropriate, and any deviations from the standard RCT design (individual randomization, parallel groups) accounted for in the conduct and analysis of the trial?

**Table 3.** Quality assessment of the included quasi-experimental studies.

Author and year	Q1	Q2	Q3	Q4	Q5	Q6	Q7	Q8	Q9	Score/9
Barbosa-Torres 2021^44^	Y	Y	Y	N	Y	Y	Y	Y	N	7/9
Lazaridou 2019^51^	Y	Y	Y	N	Y	Y	Y	Y	N	7/9
Romero-Zurita 2012^61^	Y	Y	N	N	Y	Y	Y	Y	N	6/9
Sawynok 2013^63^	Y	Y	Y	N	Y	Y	Y	Y	N	7/9
Segura-Jimenez 2014^65^	Y	Y	Y	N	Y	Y	Y	Y	N	7/9

Abbreviations: Y, yes; N, no.

1. Is it clear in the study what is the “cause” and what is the “effect” (ie, there is no confusion about which variable comes first)?

2. Were the participants included in any comparisons similar?

3. Were the participants included in any comparisons receiving similar treatment/care, other than the exposure or intervention of interest?

4. Was there a control group?

5. Were there multiple measurements of the outcome both pre and post the intervention/exposure?

6. Was follow-up complete and if not, were differences between groups in terms of their follow-up adequately described and analyzed?

7. Were the outcomes of participants included in any comparisons measured in the same way?

8. Were outcomes measured in a reliable way?

9. Was appropriate statistical analysis used?

### Analysis of findings

In the following paragraphs, we provide a narrative overview of the outcomes related to pain, fatigue, PGIC, multidimensional function, sleep disturbance, depression, anxiety, and AEs across the different mind-body therapies. A comprehensive summary of our findings on these patient-important outcomes is presented in Table 4. Additional health-related outcomes reported in the included studies are detailed in Table 5.

**Table 4.** Main outcomes and findings of included studies (relevant patient-important outcomes).

Author and year	Type of mind-body therapy	Intensity and frequency	Control	Patient-important outcomes	Relevant instruments	Duration and follow-up	Main findings (intervention vs control arm)	Challenges encountered	Conclusion
Alba 2022^42^; Terrasa 2020^67^	Biofeedback	SMR NFB training 3 sessions per week. NFB tasks included moving a ball to a computerized target.	The attention control intervention was false feedback (sham).	Pain, fatigue, multidimensional function (FM impact), and anxiety	SF-36 Pain subscale (pain), WHYMPI (pain), MPQ (pain), PVAQ (pain), SF-36 Vitality subscale (fatigue), FIQ (multidimensional function), BDI (depression), PASS (anxiety), and STAI (anxiety)	2 weeks Measurements pre-test and post-test	Positive: Good-SMR responders had significantly higher scores than poor SMR responders on SF-36 (pain) (*P* < .05) post-intervention. No follow-up assessments AEs not reported	Study Design: Small sample size Lack of control for quality of reinforcement during training potentially influenced SMR self-regulation Study Population: Potential bias in the results due to participants taking regular medication during NFB training	SMR NFB training showed significant improvements in FM function and symptoms including pain.
Andres-Rodriguez 2019^43^	MBSR	Usual care + weekly MBSR instructor-led training sessions once a week for 2 hours. Participants were told to practice mindfulness for 45 minutes daily at home with guidance from workbooks and audio CDs and participate in an intensive mindfulness meditation retreat for 6 hours.	The usual care was pharmacological treatment and counselling on aerobic exercise.	Multidimensional impact (FM impact), depression, and anxiety	FIQR (multidimensional function), HADS-D (depression), and HADS-A (anxiety)	8 weeks Measurements pre-test, posttest, and follow-up 12 months	Positive: Significant improvement in FIQR (multidimensional function) (*P* = .005); HADS-D (depression) (*P* = .006) post-intervention; and insignificant changes in anxiety. Follow-up assessments not reported AEs not reported	Study Design: Small sample size Study could not completely control for patients taking antidepressants Potential immune regulatory effects due to ethical reasons	MBSR intervention significantly improved symptoms and clinical severity of FM.
Barbosa-Torres 2021^44^	Biofeedback	SMR NFB training sessions 3 times per week for 15 minutes each. Sessions involved completing puzzles with subsequent rewards that included puzzle pieces and auditory beeps with auditory and visual stimuli.	A control group was not utilized.	Pain intensity, multidimensional function (FM impact, general health), depression, and anxiety	VAS (pain intensity), FIQR and GHQ-28 (multidimensional function), GHQ-28 (depression and anxiety)	7 weeks Measurements pre-test and post-test	Positive: Significant improvement in VAS (pain intensity) (*P* < .001) post-intervention; FIQR (multidimensional function) (*P* < .001) post-intervention, GHQ-28 (overall general health) (*P* = .011) post-intervention; depression (*P* < .001) post-intervention; and anxiety (*P* < .001) post-intervention. No follow-up assessments AEs not reported	Study Design: Small sample size Condition A4 of the brain state (assessing cognitive effort) of SMR training required a lot of cognitive resources and limited task concentration and improvements Small number of NFB training sessions	SMR NFB intervention significantly improved impact of symptoms of FM.
Baumueller 2017^45^	Biofeedback	EMG biofeedback instructor-led sessions 3 times a week for 3 weeks, then 1 session per week for 5 weeks. Participants were told to apply 3 minutes of conscious strain on the trapezius muscles and relax for 10 minutes. They were also instructed to follow an exercise program about conscious muscle relaxation for 15 minutes daily at home and during stressful events.	Usual care (not specified).	Pain, fatigue, patient-rated global, multidimensional function, and depression	SF-36 Pain subscale (pain); SF-36 Vitality subscale (fatigue), PGIC (patient-rated global), FIQ and SF-36 (multidimensional function), and BDI (depression)	8 weeks Measurements pre-test, post-test, and follow-up 3 months	No significant improvement in pain, fatigue, patient-rated global, multidimensional function or depression post-intervention or at follow-up. AEs not reported	Study Design: Small sample size Lack of patient blinding Lack of a sham control group due to recognition of sham treatment by patients Lack of verification for adherence to the home exercise program for muscle relaxation	No significant improvements observed among FM patients after EMG biofeedback intervention.
Carson 2012^46^	Yoga	Yoga of Awareness (YoA) sessions once a week for 120 minutes. Sessions included gentle stretching for 40 minutes, meditation for 25 minutes, breathing techniques for 10 minutes, presentations including yoga principles and optimal coping for 20 minutes, and group discussions for 25 minutes. Participants were also told to practice certain yoga techniques for 20–40 minutes each day for 5–7 days weekly at home using DVDs and CDs.	The wait-list group underwent routine FM care between the baseline and the second assessment time point.	Pain, fatigue, patient-rated global, multidimensional function, sleep disturbance, depression, and anxiety	FIQR Pain subscale (pain), FIQR Fatigue subscale (fatigue), PGIC (patient-rated global), FIQR (multidimensional function), FIQR Poor Sleep subscale (sleep disturbance), FIQR Depression subscale (depression), and FIQR Anxiety subscale (anxiety)	8 weeks Measurements pre-test, post-test, and follow-up 3 months	Insignificant changes observed in FIQR (pain) post-intervention and at follow-up; FIQR (fatigue) post-intervention and at follow-up; FIQR (multidimensional function) post-intervention and at follow-up; depression post-intervention and at follow-up; and anxiety post-intervention and at follow-up. AEs not reported	Study Design: Small sample size Lack of active control group to control for attention biases or placebo effect from intervention Significant reliance on self-reported data Potential effects from therapist due to the singular intervention provider Study Population: Majority middle-class educated white individuals Potential selection bias due to previous interest in study	YoA intervention provides improvements trending towards clinical significance in treating FM symptoms and various factors with potentially larger benefits following more practice sessions.
Cash 2015^47^	MBSR	MBSR instructor-led sessions once a week for 2.5 hours. The instructors taught attention-focusing techniques such as body scanning, sitting meditation, and yoga positions for relaxation. Participants were told to practice 45 minutes per day for 6 days per week at home with assistance from workbooks and audio tapes. A meditation retreat was presented for half a day between week 6 and 7.	The wait-list group was presented with the MBSR program after the study ended.	Pain, fatigue, and sleep disturbance	VAS (pain), FSI (fatigue), and SSQ (sleep disturbance)	8 weeks Measurements pre-test, post-test, and follow-up 2 months	Positive: Significant improvement in FSI (fatigue) (*P* = .002) post-intervention but did not persist at follow-up; and SSQ (sleep disturbance) (*P* = .038) at post-intervention and follow-up, however when excluding participants lost to follow-up, sleep improvements were no longer significant. Insignificant improvements observed in VAS (pain) post-intervention or at follow-up Greater improvements were correlated with greater at-home practice AEs not reported	Study Design: High physical function scores limited detection of improvement from intervention Symptom severity in patients limited generalizability to healthier patients Study Population: Racially similar patients limited generalizability Patient-reported mindfulness not measured due to previous lack of validated mindfulness measures	MBSR intervention significantly improved sleep disturbance.
Grossman 2017^48^; Schmidt 2011^66^	MBSR	Standard MBSR sessions once a week for 2.5 hours.	A wait-list group was utilized.	Pain perception, multidimensional function (FM impact and HRQoL), sleep disturbance, depression, and anxiety	PPS (pain perception), FIQ and PLC (multidimensional function), PSQI (sleep disturbance), CES-D (depression), and STAI (anxiety)	8 weeks Measurements pre-test, post-test, and follow-up 8 weeks	No significant improvements in primary outcome, HRQoL, at post-intervention or follow-up in comparison to wait-list procedure. However, the MBSR group displayed significant improvements in 6 of 8 secondary outcomes post-intervention and at follow-up, including FIQ (FM impact) (*P* = .021); CES-D (depression) (*P* = .012); STAI (anxiety) (*P* = .003); PSQI (sleep quality/disturbance) (*P* = .004); PSS (pain perception) (affective pain, *P* < .001); and GCQ (physical symptoms) (*P* < .001). The wait-list group exhibited improvement in 2 outcomes including affective pain perception (*P* = .026) and complaints (*P* = .025).	Study Design: Some participants found study to be uncomfortable and refused to participate which potentially influenced results Size of study limited monitoring to single day for each measuring date Greater monitoring past daytime raises privacy issues Lack of between-group comparison with control group for majority of outcomes	MBSR had significant within-group improvements in outcomes including pain, multidimensional function, depression, anxiety, sleep disturbance, and physical symptoms. Nevertheless, since enhancements were not assessed in comparison to the control group, it is not possible to assert that MBSR produced the anticipated effects and outperformed the wait-list procedure.
Jones 2012^49^	Tai chi	FM-modified 8-form Yang-style tai chi 2 sessions per week for 90 minutes. The sessions emphasized slow, gentle, controlled/rhythmic movements during exercise, self-massage, natural breathing, and relaxation. The postures were static and dynamic. The exercises consisted of warming up for 15 minutes, tai chi for 45 minutes, a break for 15 minutes, and a cool-down period for 15 minutes.	The control group consisted of 90-minute group sessions for 2 sessions/week focusing on FM facts (week 1), healthy eating (weeks 3-7) and psychoeducation about FM (8-11).	Pain, fatigue, multidimensional function, sleep disturbance, depression, and anxiety	FIQ and BPI (pain), FIQ Fatigue subscale (fatigue), FIQ (multidimensional function), PSQI and FIQ Sleep subscale (sleep disturbance), FIQ non-pain symptoms subscale (depression), and FIQ non-pain symptoms subscale (anxiety)	12 weeks Measurements pre-test and post-test	Positive: Significant improvements in FIQ (pain) (*P* = .0000) post-intervention, BPI (severity) (*P* = .0008) post-intervention, BPI (interference) (*P* = .0000) post-intervention; FIQ Fatigue subscale (fatigue) (*P* = .0001) post-intervention; FIQ total scores (multidimensional function) (*P* = .0002) post-intervention; FIQ Sleep subscale (sleep disturbance) (*P* = .0001) post-intervention, PSQI (sleep disturbance) (*P* = .0003) post-intervention; FIQ non-pain symptoms subscale (depression) (*P* = .0001) post-intervention; and FIQ non-pain symptoms subscale (anxiety) (*P* = .0001) post-intervention. No follow-up assessments AEs measured but no events	Study Design: Difficult to develop double-blind study due to lack of sham tai chi treatment Potential placebo effect from single-blind nature of study Study Population: Higher levels of education Decreased ethnic diversity in comparison to previous studies Lack of children and sufficient number of men to study gender differences Lack of follow-up contrary to previous studies	12-week 8-form tai chi significantly improved FM symptoms including pain, fatigue, multidimensional function, sleep, anxiety, and depression compared to education control with potential for long-term improvements. Results demonstrate significance of tai chi as additional treatment to FM.
Kaplun 2021^50^	Guided Imagery	Guided imagery sessions once a week for 1 hour. Training comprised 6 techniques that emphasized breathing, reducing pain and suffering through the tree, olive oil, magnet, blue light, and the waterfall exercises. Duration per exercise was 1–2 minutes. The participants were told to perform 3 brief guided imagery exercises per day.	The wait-list group was presented with guided imagery therapy after the intervention period ended.	Pain, fatigue, and sleep disturbance	SF-36 Pain subscale (pain), BPI (average, mildest and overall pain), SF-36 Energy/fatigue subscale (fatigue), and BPI Sleep subscale (sleep disturbance)	6 weeks Measurements pre-test and post-test	Positive: Significant improvement observed in SF-36 Pain subscale (pain) (*P* = .000) post-intervention, BPI (average pain) (*P* = .011) post-intervention, BPI (mildest pain) (*P* = .003) post-intervention, BPI (overall pain) (*P* = .003) post-intervention; SF-36 Energy/fatigue subscale (fatigue) (*P* = .006) post-intervention; and BPI Sleep subscale (sleep disturbance) (*P* = .025) post-intervention. Significant increases in all areas except relief due to medication, physical/social functioning and role limitation due to physical health. No follow-up assessments AEs not reported	Study Design: Small sample size leading to potential for self-selection bias Only 1 therapist throughout experiment Potential benefits from group therapy sessions/interactions instead of intervention Lack of monitoring outcomes during intervention Lack of follow-up Timing of study prevented some eligible individuals from partaking	Brief guided imagery significantly improved pain, fatigue, and sleep disturbance. Suggests the neurological processes during brain practice in brief guided imagery should be analyzed in future studies.
Lazaridou 2019^51^	Yoga	Satyananda yoga instructor-led in-person sessions once a week for 1.5 hours. Meditation, asanas, and mindfulness-based practices were taught, and daily 30-minute yoga videos were provided to follow at home that guided asanas, meditation, and breathing exercises.	There is no control group in this study.	Pain, fatigue, multidimensional function, (FM impact) sleep disturbance, and anxiety	BPI (pain), daily diary (fatigue), FIQR (multidimensional function), PSQI (sleep disturbance), and PROMIS (anxiety)	6 weeks Measurements pre-test and post-test	Positive: Significant improvement in BPI (pain) (*P* = .041) post-intervention; fatigue (*P* = .003) post-intervention; and PSQI (sleep disturbance) (*P* = .027) post-intervention. Insignificant changes in anxiety and multidimensional function post-intervention. No follow-up assessments AEs not reported	Study Design: Small sample size Lack of control group limiting generalizability Multiple steps of intervention limit clarity in which component was beneficial Study Population: High variability between patients and symptoms Voluntary participation potentially leading to selection bias High initial drop-out rate suggests great difficulty of intervention	Daily yoga-based exercise positively impacted pain, fatigue, and sleep among individuals with FM with a greater effect seen among those who engaged in more at-home practice. Future studies should look towards examining specific components of the intervention that target symptoms of FM most effectively.
Liu 2012^52^	Qi gong	The “six healing sound” instructor-led qi gong sessions once a week for 45-60 minutes. The qi gong exercises were completed in sitting, standing, and supine body postures. Practicing at home was encouraged twice per day for 15-20 minutes.	The attention control intervention was the sham treatment, consisting of similar movements without meditation or healing sounds, twice per day at home for 15-20 minutes.	Pain, fatigue, multidimensional function (FM impact), and sleep disturbance	SMPQ (pain), MFI-20 (fatigue), FIQ (multidimensional function), and PSQI (sleep disturbance)	6 weeks Measurements pre-test and post-test	Positive: Significant improvements include decrease in SMPQ (pain) (*P* < .0125) post-intervention; MFI-20 (fatigue) (*P* < .0125) post-intervention; FIQ (multidimensional function) (*P* < .0125) post-intervention; PSQI trending towards significance. No follow-up assessments AEs measured but no events	Study Design: Small sample size Lack of blinding	Qi gong significantly improved pain, fatigue, and multidimensional function. This specific form of qi gong exercise was easy to learn and practice.
Lynch 2012^53^, Sawynok 2014^64^	Qi gong	The Chaoyi Fanhuan qi gong sessions once a week for 60 minutes. The intervention consisted of 7 movements that emphasized relaxation, softness, and downward releases. Participants were instructed to practice once a day for 45-60 minutes at home.	The wait-list control group consisted of proceeding with their usual care.	Pain, multidimensional function (FM impact and quality of life), and sleep disturbance	NRS-PI (pain), FIQ (multidimensional function), and PSQI (sleep disturbance)	24 weeks Measurements pre-test, 8 weeks, 16 weeks, and post-test	Positive: Significant improvement in pain at 8 weeks (*P* < .001), 16 weeks (*P* = .01) and 24 weeks (*P* = .02); FIQ (multidimensional function) at 8 weeks (*P* < .001), 16 weeks (*P* = .003), and 24 weeks (*P* = .007); and PSQI (sleep disturbance) at 8 weeks (*P* = .001), 16 weeks (*P* < .001), and 24 weeks (*P* = .003). Qualitative comments about improved outcomes among patients Greater improvements linked to greater practice times No post-intervention follow-up assessments 2 AEs were encountered	Study Design: Lack of blinding Control group did not receive same level of attention in comparison to intervention group Variable adherence to qi gong practice potentially affected mean results Specific form of qi gong implemented reducing generalizability Difficult to determine dose–response relationship	Self-practice level 1 Chaoyi Fanhuan qi gong training provided significant long-term improvements in areas including pain, multidimensional function, and sleep disturbance. Greater improvements were associated with those that adhered and practiced as per the recommended protocol (≥ 5 hours/week) in comparison to minimal practice (≤ 3 hours/week).
Maddali Bongi 2016^54^	Tai chi	Tai ji quan sessions twice a week for 60 minutes. It consisted of 15 minutes of breathing and posture correction, 15 minutes of low-impact movements, and 30 minutes of 14 modified tai ji quan movements which were performed slowly, controlled and in a relaxed manner.	The control group consisted of educational sessions about FM syndrome and coping twice per week.	Pain, fatigue, multidimensional function (FM impact, quality of life), sleep disturbance, depression, and anxiety	SF-36 Pain subscale (pain), FACIT-F and SF-36 Vitality subscale (fatigue), FIQ (multidimensional function), PSQI and PSQI Sleep disturbance subscale (sleep disturbance), HADS-D (depression), and HADS-A (anxiety)	16 weeks Measurements pre-test and post-test	Positive: Significant improvements in SF-36 Pain subscale (pain) (*P* < .001) post-intervention; FACIT-F (fatigue) (*P* < .01) post-intervention and SF-36 Vitality subscale (fatigue) (*P* < .01) post-intervention; FIQ (multidimensional function) (*P* < .05) post-intervention; PSQI (sleep disturbance) (*P* < .05) post-intervention and PSQI Sleep disturbance subscale (sleep disturbance) (*P* = .001) post-intervention; and HADS-A (anxiety) (*P* < .05) post-intervention; insignificant changes in depression post-intervention. No follow-up assessments AEs measured but no events	Study Design: Small sample size Lack of follow-up Lack of individualized program specific to participants’ abilities	Tai ji quan therapy significantly improved pain, fatigue, multidimensional function, sleep disturbance, and anxiety. Successfully accepted by FM patients, evident by high adherence to the intervention.
Medina 2022^55^	MBSR	MBSR sessions once a week for 2 hours. Group and homework sessions consisted of MBSR practice (eg, sitting meditation, body scan, and mindful movements). Participants were also encouraged to attend a silent MBSR retreat for half a day between weeks 6 and 7.	The usual care consisted of medications with aerobic exercise counselling, based on each patient’s physical capabilities.	Pain intensity, multidimensional function (FM impact), depression, and anxiety	VAS (pain intensity), FIQR (multidimensional function), HADS (depression), and HADS (anxiety)	8 weeks Measurements pre-test and post-test	Positive: No significant improvements in VAS (pain), FIQR (multidimensional function), HADS (depression), HADS (anxiety). No follow-up assessments AEs not reported	Study Design: Small sample size Limited images of brain activity from regional cerebral blood flow maps focusing on specific areas prevented whole brain analyses of effects from interventions	Both nonpharmacological interventions demonstrated changes in connectivity between brain activity through regional cerebral blood flow and symptoms of FM such as pain catastrophizing.
Menzies 2014^56^	Guided Imagery	Instructed to listen to 3 20-minute CD tracks at least once daily (Track 1-guided relaxation for weeks 1–2, Track 2- elicit sensory involvement with pleasant scene imagery for weeks 3–4, Track 3- a guided journey through the immune system for weeks 5–6).	The control group maintained their treatment as usual.	Pain severity, pain interference, fatigue, and depression	BPI (pain severity and pain interference), BFI (fatigue), and CES-D (depression)	10 weeks Measurements pre-test, week 6, and post-test.	Positive: Significant improvements in BPI (pain severity) at week 6 (*P* = .03) and week 10 (*P* < .01); BFI (fatigue) *P* = .02) at week 6 and week 10 (*P* < .01); CES-D (depression) at week 10 (*P* = .02); and marginal significance observed in BPI (pain interference) at weeks 6 (*P* = .08) and 10 (*P* = .09). No follow-up assessments AEs not reported	Study Design: Lack of active control group Inaccurate self-reported data of at-home GI exercises Lack of explanation for mechanisms underlying the relationships between the FM parameters	Guided imagery helped to significantly improve pain intensity, fatigue, and depression. Provides evidence to implement guided imagery as treatment for managing negative FM symptoms.
Onieva-Zafra 2015^57^, 2019^58^	Guided Imagery	Three 1.5-hour guided imagery group sessions and instructions on using 2 guided imagery CDs containing 15-minute recordings of relaxation and visualization techniques. Listening instructions included 4 sessions in weeks 1 and 3, daily use in weeks 2 and 4, and independent use in weeks 5 to 8.	Attention control group intervention consisted of three 1.5-hour sessions involving group conversations and reporting of symptoms and experiences.	Pain, fatigue, multidimensional function, sleep disturbance, depression, and anxiety	VAS and MPQ-LF (pain), SF-36 Vitality subscale (fatigue), FIQ (multidimensional function), PSQI (sleep disturbance), VAS and BDI (depression), and STAI (anxiety)	8 weeks Measurements at pre-test, week 4, and post-test	Positive: A significant decrease in MPQ-LF (sensory pain) at week 4 (*P* = .039) and week 8 (*P* = .042), MPQ-LF (affective pain) at week 4 (*P* = .044), MPQ-LF (evaluative pain) at week 4 (*P* = .041) and week 8 (*P* = .044), VAS (pain) at week 4 (*P* = .048); SF-36 Vitality subscale (fatigue) at week 4 (*P* < .001) and week 8 and (*P* < .001); PSQI Sleep disturbance subscale (sleep disturbance) at week 4 (*P* = .037) and week 8 (*P* = .046); BDI (depression) at week 4 (*P* = .010) and week 8 (*P* = .001); and STAI (trait anxiety) at week 4 (*P* < .048) and week 8 (p-value not reported). No follow-up assessments AEs not reported	Validation: Small sample sizes Short intervention periods Subjective pain and depression results Patients not assessed immediately post-intervention Lack of blinding did not account for home environment, individual conditions, and social status	Guided imagery significantly improved FM symptoms such as fatigue, sleep disturbance, depression, anxiety, and the sensory, affective, and evaluative dimensions of pain.
Perez-Aranda 2019^59^	MBSR	2-hour weekly group sessions consisting of meditation exercises and audiotapes for home use.	The control group maintained treatment as usual (eg, anxiolytics, analgesics, antidepressants, opioids).	Multidimensional function (FM impact), PGIC, and depression	FIQR (multidimensional function), PGIC, HADS-Total (depression and anxiety), HADS (depression)	8 weeks Measurements pre-test, post-test, and follow-up 12 months	Positive: A significant improvement in FIQR (multidimensional function) compared to TAU post-intervention (*P* < .001) and at follow-up (*P* = .001); PGIC post-intervention (*P* < .001); and HADS (depression) post-intervention with mindfulness practice (*P* = .03), and marginal improvement at 12 months with mindfulness practice (*P* = .05). 9 AEs were encountered	Intervention efficacy: Lacking group MBSR efficacy potentially reduced adherence Lack of equal distribution led to fewer participants with major depression in MBSR Low follow-up rates	MBSR demonstrated the most significant reductions in FM-related feelings in comparison to TAU and FibroQoL.
Rodriguez-Mansilla 2021^60^	Qi gong	Qi gong sessions twice a week for 45 minutes. The qi gong exercises emphasized concentration, abdominal breathing, balance, controlled movements, and flexibility.	No intervention was provided for the control group and treatment as usual was continued.	Pain and multidimensional function (FM impact)	VAS (pain) and S-FIQ (multidimensional function)	4 weeks Measurements pre-test and post-test	Positive: A significant reduction in VAS (pain) (*P* = .001) post-intervention; and S-FIQ (multidimensional function) pos-intervention (*P* = .002). No follow-up assessments AEs not reported	Limitations: Difficulty in learning intervention exercises Short intervention period potentially impacting outcomes	Exercise for well-being (qi gong) and the active exercise program significantly improved pain, and quality of life among FM patients.
Romero-Zurita 2012^61^	Tai chi	Low intensity FM-modified 8-form Yang-style tai chi 3 sessions per week for 60 minutes. Sessions included warmups, breathing, stretching, mobility, tai chi, and relaxation techniques.	A control group was not utilized.	Pain, fatigue, multidimensional function (FM impact), depression, and anxiety	SF-36 Pain subscale (pain), VAS (pain), FIQ VAS Fatigue subscale and SF-36 Vitality subscale (fatigue), FIQ (multidimensional function), VAS and HADS (depression), and VAS and HADS (anxiety)	28 weeks Measurements pre-test, post-test, and 12-week detraining period	Positive: A significant improvement in VAS (pain) post-intervention (*P* < .001), SF-36 Pain subscale (pain) post-intervention (*P* = .003) and after the 12-week detraining period (p-value not reported); FIQ VAS Fatigue subscale (fatigue) post-intervention (*P* < .001), SF-36 Vitality subscale (fatigue) post-intervention (*P* = .018) and detraining (p-value not reported); FIQ (multidimensional function) post-intervention (*P* < .001); VAS (depression) post-intervention (*P* < .001), HADS (depression) post-intervention (*P* < .001); VAS (anxiety) post-intervention (*P* < .001), HADS (anxiety) post-intervention (*P* = .009); insignificant improvement observed after the 12-week detraining period in VAS (pain), VAS (depression), VAS (anxiety), HADS (depression) and HADS (anxiety). AEs measured but no events	Difficult to compare results with other studies as this is the first study to analyze long-term effects of tai chi among FM patients No RCT with control group Unable to keep FM pharmacological treatments consistent during intervention Unable to control preexisting notions about tai chi	Modified 8-Form Yang Style tai chi resulted in significant improvements in symptomatology, quality of life, functional capacity, and psychological outcomes among FM patients.
Sarmento 2020^62^	Qi gong	Group qi gong sessions 1 time per week for 45 minutes. At home “Six healing sounds” qi gong sessions twice per day for 25 minutes. Qi gong exercises included deep breathing synchronized with mild body movements, and meditation while saying six “healing sounds.”	Sham qi gong was conducted with similar body movements excluding healing sounds, meditation and diaphragmatic breathing.	Pain, fatigue, multidimensional function (FM impact and quality of life), sleep disturbance, depression, and anxiety	VAS and SMPQ (pain), FIQR VAS Fatigue subscale (fatigue), FIQR and QOLS (multidimensional function), PSQI (sleep disturbance), HADS (depression), and HADS (anxiety)	10 weeks Measurements pre-test and post-test	Positive: A significant decrease in SMPQ (*P* < .01) post-intervention, VAS (pain) (*P* < .05) post-intervention; FIQR VAS Fatigue subscale post-intervention (*P* < .05); FIQR (multidimensional function) post-intervention (*P* < .01), QOLS (multidimensional function) (*P* < .05); PSQI (sleep disturbance) post-intervention (*P* < .01); HADS depression (*P* < .05) post-intervention; and HADS anxiety (*P* < .05) post-intervention. No follow-up assessments AEs measured but no events	Limitations: Small sample sizes Potential exposure to exercises similar to intervention Subjective outcome measures used Age differences among study groups	Qi gong significantly improved chronic fatigue, widespread pain, quality of sleep, FM intensity, anxiety and depression.
Sawynok 2013^63^	Qi gong	Weekly group qi gong sessions for 60 minutes and daily at home practice for 60 minutes. Qi gong sessions included Level 1 Chaoyi Fanhuan movements and Level 2 Chaoyi Fanhuan meditation including sitting, standing, lying meditation, and slow/rhythmical movements.	A control group was not utilized.	Pain, multidimensional function (FM impact), sleep disturbance, and patient-rated global	NRS-PI (pain), FIQ (multidimensional function), PSQI (sleep disturbance), and PGIC (patient-rated global)	24 weeks Measurements pre-test, week 8, week 16, post-test	Positive: Significant improvement in pain at week 8 (*P* = .010), week 16 (*P* = .028), and week 24 (*P* = .012); FIQ (multidimensional function) at week 8 (*P* = .040), week 16 (*P* = .019) and week 24 (*P* = .036); and PSQI (sleep disturbance) at week 8 (*P* = .045), week 16 (*P* = .002) and week 24 (*P* = .004). No follow-up assessments 10 AEs were encountered	Limitations: Unable to conduct blinding High variability in qi gong practices hindered associations between treatment and outcomes Difficult to determine dose–response relationship	Qi gong resulted in Significant improvements in pain, FM impact, sleep impairments, and function.
Segura-Jimenez 2014^65^	Tai chi	Modified low-moderate intensity 8-Form, Yang Style group tai chi sessions 3 times per week for 60 minutes. The tai chi sessions included posture work, slow and steady movements.	A control group was not utilized.	Pain	VAS (pain)	24 weeks Measurements pre-session and post-session	Positive: A significant cumulative change in pain at the beginning of week 16 (*P* < .001); and insignificant cumulative change in pain for the first 12 weeks. No follow-up assessments AEs measured but no events	Validation: Unable to compare results with studies due to methodological differences Limitations: Small sample size of men compared to women	Tai chi significantly improved acute pain in both 12- and 24-week intervention periods and improved cumulative pain in the 24-week intervention.
Van Gordon 2017^68^	MAT	Weekly group meditation awareness sessions for 120 minutes each.	Cognitive behavioral theory for groups. Weekly sessions consisted of a 45-minute teaching component, 30-minute group discussions, and 30-minute guided discovery educational exercises. Emphasis placed on education rather than practice or meditation.	Pain, multidimensional function (FM impact), and sleep disturbance	SFMPQ (pain), FIQR (multidimensional function), and PSQI (sleep disturbance)	8 weeks Measurements pre-test, post-test, and follow-up 24 weeks	Positive: A significant improvement in SFMPQ (pain) post-intervention (*P* < .001) and at week 24 (*P* < .001); FIQR (multidimensional function) post-intervention (*P* < .001) and follow-up (*P* < .001); and PSQI (sleep disturbance) post-intervention (*P* < .001) and follow-up (*P* < .001). AEs not reported	Limitations: Utilized subjective self-reported measures Assessments conducted only at 3 time points Potential pre-existing ideas about mindfulness/meditation	MAT significantly improved FM symptoms and perceived pain through decreased self-attachment.
Verkaik 2014^69^	Guided Imagery	Two 1.5-hour group guided imagery sessions followed by daily at home guided imagery practice sessions for approximately 20–30 minutes.	The attention control group consisted of 2 group sessions (1.5 hours each) with group discussions.	Pain intensity and multidimensional function (FM impact)	VAS (pain intensity), and FIQ (multidimensional function)	4 weeks Measurements pre-test, post-test, and follow-up 6 weeks	Insignificant changes in pain intensity post-intervention, and unspecified pain intensity results at the 6-week follow-up. Insignificant changes in FIQ (multidimensional function). AEs not reported	Intervention: Different guided imagery exercises produced varying outcomes Limitations: Only measured general pain intensity at the end of the day and not during/after exercises Direct reference to pain and FM adversities with lesser focus on positive imagery Short intervention period Sole focus on pain and not symptoms of FM, opposing participant recommendations	No significant effects of guided imagery were determined.
Wong 2018^70^	Tai chi	Supervised tai chi sessions 3 times per week for 55 minutes at 40–50% of the patient's heart rate r reserve.	The control group maintained their usual care (eg, medications, and dietary patterns).	Pain, fatigue, and sleep disturbance	VAS (pain), VAS (fatigue), and VAS (sleep disturbance)	12 weeks Measurements pre-test and post-test	Positive: A significant reduction in VAS (pain) (*P* = .006) post-intervention; VAS (fatigue) post-intervention (*P* = .001); and no significant changes were observed for sleep. No follow-up assessments AEs measured but no events	Limitations: Baroreflex sensitivity, blood pressure, and catecholamines not measured unlike previous studies Small sample size Limited generalizability due to specific age range Potential Hawthorne effect that is unaccounted for.	Tai chi training over 12 weeks had significant improvements in pain and fatigue.
Wu 2021^71^	Biofeedback	NFB training sessions 2–3 times per week for 30 minutes. SMR and alpha rhythm feedback through relaxing and focusing on a computer game.	The attention control group received weekly telephone support and educational content on FM.	Pain, multidimensional function, and sleep disturbance	BPI (pain), FIQR (multidimensional function), and PSQI (sleep disturbance)	8 weeks Measurements pre-test and post-test	Positive: A significant improvement in BPI (pain severity) post-intervention (*P* = .002), BPI (pain interference) post-intervention (*P* < .001); and FIQR total scores (multidimensional function) post-intervention (*P* = .001). No follow-up assessments AEs not reported	Limitations: EEG data not collected, preventing findings about relation between FM symptoms and EEG changes Difficult to determine dose–response relationship as not all participants completed treatment sessions Long-term effects not examined	NFB training via SMR and alpha waves significantly alleviated FM symptoms and pain.
Yoo 2022^72^	PMR	Group PMR sessions twice per week for 40 minutes. Participants were also instructed to perform exercises at home twice per day for 40 minutes.	The attention control group underwent conventional physical therapy and then received the intervention tape along with 4 40-minute PMR sessions for 1 week.	Pain and fatigue	VAS (pain), and MAF (fatigue)	8 weeks Measurements pre-test and post-test	Positive: A significant improvement in VAS (pain) post-intervention (*P* < .001); and in MAF (fatigue) post-intervention (*P* < .001). No follow-up assessments AEs not reported	Limitations: Did not examine long-term effects of intervention Seasonal changes in temperature and humidity with effects on participants unaccounted for Participant characteristics not measured Small sample size Limited use of tools to determine pain expression	PMR significantly improved pain and fatigue in patients with FM.

Abbreviations: AE, Adverse event; BDI, Beck Depression Inventory; BFI, Brief Fatigue Inventory; BPI, Brief Pain Inventory; CES-D, Center for Epidemiological Studies-Depression scale; EEG, Electroencephalogram; EMG, Electromyogram; FACIT-F, Functional Assessment of Chronic Illness Therapy-Fatigue Scale; FibroQoL, Multicomponent intervention for Fibromyalgia; FIQ, Fibromyalgia Impact Questionnaire; FIQR, Fibromyalgia Impact Questionnaire Revised; FM, Fibromyalgia; FSI, Fatigue Symptom Inventory; GCQ, Giessen Complaint Questionnaire; GHQ-28, General Health Questionnaire; HADS, Hospital Anxiety and Depression Scale (A-anxiety subscale; D-depression subscale); HRQoL, Health-related quality of life; MAF, Multidimensional Assessment of Fatigue; MAT, Meditation Awareness Training; MBSR, Mindfulness-Based Stress Reduction; MFI-20, 20-item Multidimensional Fatigue Inventory; MPQ, McGill Pain Questionnaire; MPQ-LF, McGill Pain Questionnaire Long Form; NFB, Neurofeedback; NRS-PI, 11-point numerical rating scale for pain intensity; PASS, Pain Anxiety Symptoms Scale; PGIC, Patient Global Impression of Change; PLC, Quality of Life Profile for the Chronically Ill; PMR, Progressive Muscle Relaxation; PPS, Pain Perception Scale; PROMIS, Patient-Reported Outcomes Measurement Information System; PSQI, Pittsburgh Sleep Quality Index; PVAQ, Pain Vigilance and Awareness Questionnaire; QOLS, Quality of Life Scale; SF-36, 36-item Short-Form Health Survey; S-FIQ, Spanish Fibromyalgia Impact Questionnaire; SMPQ/SFMPQ, Short-Form McGill Pain Questionnaire; SMR, Sensorimotor Rhythm; SSQ, Stanford Sleep Questionnaire; STAI, State-Trait-Anxiety-Inventory; TAU, Treatment-as-usual; VAS, Visual analogue scale; WHYMPI, West Haven-Yale Multidimensional Pain Inventory; YoA, Yoga of Awareness.

**Table 5.** Other health-related outcomes and findings of included studies.

Author and year	Type of mind-body therapy	Other health-related outcomes	Relevant instruments	Related findings
Alba 2022^42^; Terrasa 2020^67^	Biofeedback	QoL, kinesiophobia, coping strategies, social support, connectivity between central region and cortical regions of brain, and between neural activity and heart rate	SF-36 (QoL), TSK (kinesiophobia), CSQ (coping strategies), MOS (social support), fMRI (connectivity between central region and cortical regions of brain), and EEG (connectivity between neural activity and heart rate)	Positive: Good SMR responders showed improvement in SF-36 general health perception (*P* < .05), SF-36-change in health (*P* < .01), and increased functional connectivity of motor and somatosensory areas (*P* < .001) post-intervention.
Andres-Rodriguez 2019^43^	MBSR	Perceived stress, general distress, subjective cognitive function, pain catastrophizing, psychological inflexibility (avoidance and cognitive fusion related to pain), mindfulness (observing, describing, acting with awareness, non-judging of inner experience, non-reacting to inner experience), serum levels of immune biomarkers including cytokines and chemokines (pro-inflammatory Interleukin (IL)-6, CXCL8, and high-sensitivity C-reactive protein [hs-CRP] and anti-inflammatory IL-10)	PSS (perceived stress), HADS (general distress), MISCI (subjective cognitive function), PCS (pain catastrophizing), PIPS (psychological inflexibility), FFMQ (mindfulness), blood extraction (immune biomarkers)	Positive: Significantly improved PSS (perceived stress) (*P* = .006); HADS (general distress) (*P* = .020); MISCI (cognitive impairment) (*P* = .012); PCS (pain catastrophizing) (*P* = .036); FFMQ (mindfulness) (*P* < .001); and helped maintain beneficial anti-inflammatory cytokine levels of (IL)-10 (*P* = .034) post-intervention.
Barbosa-Torres 2021^44^	Biofeedback	SMR NFB	EEG on the sensorimotor cortex on right side of the scalp (SMR feedback, theta waves)	Positive: Significant increase in amplitude of SMR waves (*P* = .010), decrease in theta waves (*P* < .001), and increase in ratio of SMR/theta waves (*P* < .001).
Baumueller 2017^45^	Biofeedback	Psychological distress, QoL aspects, pain intensity at commonly painful tender points, and widespread pain and tenderness	SCL-90-R (psychological distress), SF-36 subscales (physical functioning, role-physical, general health, social functioning, role-emotional, mental health), TPS (pain intensity), TPC test and dolorimeter (widespread pain and tenderness)	No significant improvements in health status. Positive: Significant improvement solely in pain-pressure threshold in the trapezius muscle post-intervention (*P* = .016).
Carson 2012^46^	Yoga	Pain in myalgic tender points, strength, balance, pain acceptance, pain catastrophizing, pain coping strategies, and daily variables	TMS (pain in myalgic tender points) Timed Chair Rise (strength), SCBT (balance), CPAQ (pain acceptance), CSQ (pain catastrophizing), VMPCI (pain coping), and daily diaries (daily variables including pain, fatigue, distress, vigor, relaxation, and acceptance)	Positive: VMPCI use of religion subscale post-intervention (*P* = .007); and daily diaries Daily acceptance subscale post-intervention (*P* = .006).
Cash 2015^47^	MBSR	Perceived stress, symptom severity, physical functioning, and neuroendocrine function	PSS (perceived stress), FIQ symptom severity (symptom severity), FIQ physical functioning (physical functioning), and salivary cortisol (neuroendocrine function)	Positive: Significant improvement in PSS (perceived stress) (*P* = .000); and FIQ symptom severity (*P* = .012) at post-intervention and follow-up. No significant improvements were found in pain, physical functioning, or cortisol.
Grossman 2017^48^; Schmidt 2011^66^	MBSR	Mindfulness, daily experiences, ambulatory accelerometry, and cardiorespiratory function (respiratory sinus arrhythmia and ventilation)	FMI (mindfulness), daily diaries (daily experiences), accelerometer (ambulatory accelerometry and physical activity), and ECG and inductance plethysmography bands (cardiorespiratory function and respiration)	No significant effects from MBSR intervention were reported.
Jones 2012^49^	Tai chi	Self-efficacy, functional mobility, dynamic balance, static balance, and upper extremity flexibility	ASES (self-efficacy), TUG test (functional mobility), Maximum reach test (dynamic balance), SLS (static balance), and external/internal rotation of shoulders (upper extremity flexibility)	Positive: Significant improvements at post-intervention in ASES (self-efficacy) (*P* = .0001); TUG (functional mobility) (*P* = .0001); SLS (static balance) (*P* = .0001); and Maximum reach test (dynamic balance) (*P* = .0001).
Kaplun 2021^50^	Guided Imagery	Pain and QoL	BPI (overall activity, mood, walking ability, routine work, relationships with others, enjoyment of life, relief due to medication) and SF-36 (physical/social functioning, role limitation due to emotional/physical health, emotional wellbeing, and overall health)	Positive: Regarding BPI, significant improvements in overall activity (*P* = .002), mood (*P* = .001), walking ability (*P* = .006), and enjoyment of life (*P* = .025) post-intervention. Regarding SF-36, significant improvements in physical functioning (*P* = .030), role limitation due to emotional health (*P* = .046), emotional wellbeing (*P* = .007), and overall health (*P* = .001) post-intervention.
Lazaridou 2019^51^	Yoga	Pain catastrophizing, sleep actigraphy, and ecological momentary assessments	PCS (pain catastrophizing), wrist actigraph (sleep actigraphy), and daily diaries (ecological momentary assessments)	Positive: Significant improvement in PCS (pain catastrophizing) (*P* = .039) post-intervention. No significant improvements in sleep actigraphy.
Liu 2012^52^	Qi gong	Not Available	Not Applicable	Not Applicable
Lynch 2012^53^; Sawynok 2014^64^	Qi gong	QoL	SF-36 Physical and SF-36 Mental (QoL)	Positive: Significant improvement in SF-36 Physical (physical function) at 8 weeks (*P* < .001), 16 weeks (*P* = .001), and 24 weeks (*P* = .004); and SF-36 Mental (mental function) at 8 weeks (*P* = .002).
Maddali Bongi 2016^54^	Tai chi	QoL, patient disability with rheumatic disease, sleep quality, general distress, widespread pain, and tenderness	SF-36 subscales (QoL), HAQ (patient disability with rheumatic disease), PSQI subscales (sleep quality), HADS (general distress), WPI (widespread pain), and tender points (tenderness)	Positive: Significant improvements post-intervention SF-36 (QoL) Summary physical index subscale (*P* < .05), Physical functioning, Role- physical subscale, and Role-emotional subscale (all *P* < .01), General health subscale (*P* < .001); PSQI (sleep quality) Sleep duration subscale (*P* = .01); HADS (general distress) (*P* < .05); WPI (widespread pain) (*P* < .01); and tender points (tenderness) (*P* < .0001).
Medina 2022^55^	MBSR	Pain catastrophizing, and changes in regional cerebral blood flow	PCS (pain catastrophizing) and fMRI arterial spin labeling (changes in regional cerebral blood flow)	Positive: At baseline, positive correlation between changes in regional cerebral blood flow in anterior insula and anterior cingulate cortex (ACC) with pain. Significant improvement in PCS total (pain catastrophizing) (*P* = .015) and PCS magnification (*P* = .018) post-intervention.
Menzies 2014^56^	Guided Imagery	Self-efficacy, perceived stress, and plasma levels of immune biomarkers (C-reactive protein [CRP] and cytokine levels)	ASES and OSE (self-efficacy), PSS (perceived stress), and blood plasma samples (immune biomarkers of CRP and cytokine levels)	Positive: Significant improvements in OSE (self-efficacy) (*P* = .02) at week 10 and significant improvement in PSS (stress) (*P* = .05) at week 10. No significant changes in pro-and anti-inflammatory cytokines or CRP after intervention.
Onieva-Zafra 2015^57^, 2019^58^	Guided Imagery	Pain intensity, sleep quality, self-efficacy, and QoL	Pressure algometer (pain intensity), PSQI (sleep quality), CPSS (self-efficacy), and SF-36 (QoL)	Positive: Pain intensity: Significant improvement in pressure algometry in lower cervical (left side) (*P* = .034), second left rib (*P* < .039), and left greater trochanter (*P* = .042), right gluteal muscle (*P* = .007), and left gluteal muscle (*P* = .010) at week 4. Significant improvement in pain observed for lower cervical (left side) (*P* = .017), right gluteal muscle (*P* = .039), and left gluteal muscle (*P* = .004) at week 8. Sleep quality: Significant differences between groups in PSQI for sleep latency at week 4 (*P* = .039), sleep duration at week 4 (*P* = .019) and week 8 (*P* = .038), and habitual sleep efficiency at week 4 (*P* = .045). The quality of sleep significantly improved relative to baseline (*P* < .042). Self-efficacy: Significant differences in CPSS were found between groups at week 4 (*P* < .0349) and week 8 (*P* < .001). QoL: Significant differences found in SF-36 for mental health at week 8 (*P* = .028), and between groups for role physical at week 4 (*P* < .001) and week 8 (*P* < .007), and physical function at week 4 (*P* < .005) and week 8 (*P* < .001).
Perez-Aranda 2019^59^	MBSR	FM symptom severity, pain catastrophizing, stress, cognitive impairment, mindfulness, compassion, psychological inflexibility, pain-specific impression of change, and treatment expectancy and credibility	FSDC (FM symptom severity), PCS (pain catastrophizing), PSS (stress), MISCI (cognitive impairment), FFMQ (mindfulness), SCS-12 (compassion), PIPS (psychological inflexibility), PSIC (pain-specific impression of change), and CEQ (treatment expectancy and credibility)	Positive: Symptom severity: Significant improvement in FSDC versus FibroQoL at follow-up (*P* = .001), and versus TAU at post-intervention (*P* < .001) and follow-up (*P* < .001). Pain catastrophizing: Significant improvement in PCS versus FibroQoL at post-intervention (*P* = .025) and follow-up (*P* = .015), and versus TAU at post-intervention (*P* < .001) and follow-up (*P* = .002). Stress: Significant improvement in stress in PSS versus FibroQoL at post-intervention (*P* = .001), and versus TAU at post-intervention (*P* < .001) and follow-up (*P* = .022). Cognitive Impairment: Significant improvement in MISCI versus FibroQoL at post-intervention (*P* < .001), and versus TAU at post-intervention (*P* < .001) and follow-up (*P* < .001). Mindfulness: Significant improvement in FFMQ observe versus FibroQoL at post-intervention (*P* = .002) and versus TAU at post-intervention and follow-up (*P* < .001); in describe versus FibroQoL at follow-up (*P* = .029) and versus TAU at follow-up (*P* = .004); in act with awareness versus TAU at post-intervention (*P* = .002) and follow-up (*P* = .026); in nonjudgement versus FibroQoL at post-intervention (*P* = .009) and versus TAU at post-intervention (*P* < .001) and follow-up (*P* = .005). Compassion: Significant improvement in SCS-12 versus FibroQoL at post-intervention (*P* = .050), and versus TAU at post-intervention (*P* = .009). Psychological inflexibility: Significant improvement in PIPS versus FibroQoL at post-intervention (*P* = .013) and follow-up (*P* = .037), and versus TAU at post-intervention (*P* = .001) and follow-up (*P* < .001). Pain-specific impression of change: Significant improvement in PSIC subscales post-intervention (ranging from *P* = .009 to .001). Treatment expectancy and credibility: Significant differences for CEQ subscales versus FibroQoL at post-intervention (*P* < .05).
Rodriguez-Mansilla 2021^60^	Qi gong	Static balance, center of gravity, flexibility, one-leg stance, and perceived effort made in an activity	Wii-Fit pressure platform SLS (static balance), Wii-Fit pressure platform (center of gravity), SRT (flexibility), and RPE (perceived effort made in an activity)	No significant between-group improvements in comparison to control group.
Romero-Zurita 2012^61^	Tai chi	Pain/tender points assessment, lower-body muscular strength, upper-body muscular strength, lower-body flexibility, upper-body flexibility, static balance, motor agility/dynamic balance, aerobic endurance, symptomatology, QoL, coping, global self-esteem, and self-efficacy	TPS (pain/tender points assessments), chair stand test (lower-body muscular strength), handgrip strength (upper-body muscular strength), CSR (lower-body flexibility), BS (upper-body flexibility), blind flamingo test (static balance), TUG test (motor agility/dynamic balance), 6-minute walk test (aerobic endurance), FIQ subscales (symptomatology), SF-36 subscales (QoL), VPMI (coping), RSES (global self-esteem), and GSES (self-efficacy)	Positive: Pain/tender points assessment: Significant improvements in pain threshold of tender points, tender point count and algometer score post-intervention (all *P* < .001), and detraining (p-value not reported). Lower-body muscular strength: Significant improvement in chair stand test post-intervention (*P* < .001). Upper-body muscular strength: Significant improvement in handgrip strength post-intervention (*P* = .006). Lower-body flexibility: Significant improvement in CSR post-intervention (*P* < .001). Upper-body flexibility: Significant improvement in BS post-intervention (*P* = .002). Static balance: Significant improvement in blind flamingo test post-intervention (*P* < .001). Motor agility/dynamic balance: Significant improvement in TUG test post-intervention (*P* < .001). Aerobic endurance: Significant improvement in 6-minute walk post-intervention (*P* = .006). Fibromyalgia impact/symptomatology: Significant improvement in FIQ subscales: Stiffness (*P* = .005), pain, morning tiredness post-intervention (all *P* < .001). QoL: Significant improvements in SF-36 subscales physical role, general health, physical function, social functioning, and mental health post-intervention (*P* < .001), and at detraining (excluding physical role) (p-value not reported). Coping strategies: Significant improvement in VMPI active coping post-intervention and after detraining (p-value not reported) (*P* = .019). Self-esteem: Significant improvement in RSES post-intervention (*P* = .005). Self-efficacy: Significant improvement in GSES post-intervention (*P* < .001).
Sarmento 2020^62^	Qi gong	Pressure pain threshold	Dolorimeter (pressure pain threshold)	Pain: No significant improvements in pressure pain threshold with the dolorimeter.
Sawynok 2013^63^	Qi gong	QoL (physical and mental function) and patient satisfaction	SF-36 Physical subscale (physical QoL), SF-36 Mental subscale (mental QoL), and Patient Satisfaction Scale (patient satisfaction)	Positive: Significant improvement in SF-36 physical (physical function) at week 8 (0.012, week 16 (*P* = .008), and week 24 (*P* = .004).
Segura-Jimenez 2014^65^	Tai chi	Tender points	Pressure algometer (tender points)	No significant changes reported in tender points.
Van Gordon 2017^68^	MAT	General distress, Attachment (to self, symptoms, and environment), and civic engagement	DASS (general distress), NAS (attachment), and record of work hours (civic engagement)	Positive: Significant improvement in DASS (general distress) post-intervention and follow-up (both *P* < .001); NAS (attachment) post-intervention and follow-up (both *P* < .001); and civic engagement at post-intervention (*P* < .01) and follow-up (*P* < .001).
Verkaik 2014^69^	Guided Imagery	Self-efficacy	CPSS (self-efficacy)	No significant changes reported in self-efficacy.
Wong 2018^70^	Tai chi	Heart rate variability (HRV), flexibility, muscle strength, and body composition	Cardiac autonomic modulation (HRV), SRT (flexibility), and 1RM test on leg extension machine (muscle strength), and an 8-polar tactile electrode impedance meter (body composition)	Positive: HRV: Significant decrease in sympathetic low frequency tone (LnLF, nLF) (*P* = .016), significant improvement in parasympathetic high frequency tone (LnHF, nHF) (*P* = .039), and significant decreases in sympathovagal balance (LnLF/LnHF) (*P* = .028). Flexibility: Significant improvement in the SRS post-intervention (*P* = .001). Muscle strength: Significant improvement in 1RM post-intervention (*P* = .001).
Wu 2021^71^	Biofeedback	FM function, sleep latency, and cognitive function	FIQR function, overall FIQR and FIQR symptoms (FM function), sleep onset latency in minutes (sleep latency), and PVT and DSTs (cognitive function)	Positive: Significant improvement in FIQR function (*P* = .038), and FIQR symptoms (*P* < .001) post-intervention; sleep onset latency post-intervention (*P* = .006); and improved PVT error post-intervention (*P* = .028).
Yoo 2022^72^	PMR	Perceived stress, systolic/diastolic blood pressure, heart rate, and serum cortisol levels	PSS (perceived stress), electronic sphygmomanometer (systolic/diastolic blood pressure and heart rate), and blood samples (serum cortisol levels)	Positive: Significant improvement in perceived stress post-intervention (*P* < .001); in systolic (*P* < .001) and diastolic (*P* < .05) blood pressure post-intervention, pulse rate (*P* < .001); and cortisol levels (*P* < .001) all post-intervention.

Abbreviations: 1RM, One repetition maximum; ASES, Arthritis Self-Efficacy Scale; BPI, Brief Pain Inventory; BS, Back scratch test; CEQ, Credibility/Expectancy Questionnaire; CPAQ, 20-item Chronic Pain Acceptance Questionnaire; CPSS, Chronic Pain Self-Efficacy Scale; CSQ, Coping Strategies Questionnaire; CSR, Chair sit and reach test; DASS, Depression, Anxiety, and Stress Scale; DST, Digit Span Test; ECG, Electrocardiogram; EEG, Electroencephalogram; FFMQ, Five Facets of Mindfulness Questionnaire; FibroQoL, Multicomponent intervention for Fibromyalgia; FIQ, Fibromyalgia Impact Questionnaire; FIQR, Fibromyalgia Impact Questionnaire Revised; FM, Fibromyalgia; FMI, Freiburg Mindfulness Inventory; fMRI, Functional magnetic resonance imaging; FSDC, Fibromyalgia Survey Diagnostic Criteria; GSES, General Self-Efficacy Scale; HADS, Hospital Anxiety and Depression Scale; HAQ, Health Assessment Questionnaire; MAT, Meditation Awareness Training; MBSR, Mindfulness-Based Stress Reduction; MISCI, Multidimensional Inventory of Subjective Cognitive Impairment; MOS, MOS Social Support Survey; NAS, Non-Attachment Scale; NFB, Neurofeedback; OSE, Self-efficacy for managing other symptoms; PCS, Pain Catastrophizing Scale; PIPS, Psychological Inflexibility in Pain Scale; PMR, Progressive Muscle Relaxation; PSIC, Pain-Specific Impression of Change; PSQI, Pittsburgh Sleep Quality Inventory/Pittsburgh Sleep Quality Index; PSS, Perceived Stress Scale; PVT, Psychomotor Vigilance Test; QoL, Quality of Life; RPE, Borg Scale of Perceived Exertion; RSES, Rosenberg Self-Esteem Scale; SCBT, Sensory Integration for Balance Test; SCL-90-R, Symptom Checklist 90 Revised; SCS-12, Self-Compassion Scale; SF-36, 36-item Short-form Health Survey; SLS, Single leg stance test; SMR, Sensorimotor rhythm; SRS, Sit and Reach Score; SRT, Sit and Reach Test; TAU, Treatment-as-usual; TMS, Total Myalgic Score; TPC test, Tender Point Count test; TPS, Tender Point Score; TSK, Tampa Scale for Kinesiophobia; TUG test, 8-Foot Timed Get Up and Go test; VMPCI, Vanderbilt Multidimensional Pain Coping Inventory; VPMI, Vanderbilt Pain Management Inventory; WPI, Widespread Pain Index.

### Pain

#### Mind-body therapies versus no control

All 5 studies (n = 137) investigating mind-body therapy without control groups reported significant improvements in pain post-intervention. Four of these 5 studies involved movement therapies, including yoga, tai chi, and qi gong,^51^^,^^61^^,^^63^^,^^65^ while the remaining study focused on neurofeedback.^44^ One of the 5 studies included a follow-up assessment in which significant improvement in pain levels was sustained on the 36-item Short-Form Health Survey (SF-36) but not on the Visual Analog Scale (VAS) after a 12-week detraining period.^61^ One study on tai chi showed significant improvement in pain from the 16th week, but not during the first 12 weeks.^65^ The most frequently used measurement tool was the VAS, used in 3 out of the 5 studies,^44^^,^^61^^,^^65^ while 1 study^51^ reported findings using the Brief Pain Inventory (BPI).

#### Mind-body therapies versus no specific treatment

Among 9 studies^45–47^^,^^50^^,^^53^^,^^55^^,^^56^^,^^60^^,^^64^^,^^70^ comparing mind-body therapy to no specific treatment, 5 studies (n = 287)^50^^,^^53^^,^^56^^,^^60^^,^^70^ demonstrated significant improvements in pain post-intervention. Three studies used movement therapy, including qi gong and tai chi,^53^^,^^60^^,^^64^^,^^70^ and 2 used guided imagery.^50^^,^^56^ A retrospective analysis^64^ drew from qualitative comments of participants from an original trial on qi gong.^53^ In this study, participants reported pain relief within the first 8 weeks, and some continued to experience reduced pain levels after 4–6 months of qi gong practice. The 4 remaining studies^45–47^^,^^55^ did not show significant reductions in pain post-intervention. The most commonly used measurement scale was the VAS, employed in 4 out of the 9 studies.^47^^,^^55^^,^^60^^,^^70^ This was followed by the SF-36 Pain subscale^45^^,^^50^ and the BPI,^50^^,^^56^ each used in 2 studies.

#### Mind-body therapies versus attention control interventions

Out of the 10 studies^49^^,^^52^^,^^54^^,^^57^^,^^62^^,^^67–69^^,^^71^^,^^72^ comparing mind-body therapy to an attention control group, 9 studies (n = 471) on movement therapies, including tai chi and qi gong,^49^^,^^52^^,^^54^^,^^62^ neurofeedback,^67^^,^^71^ MAT,^68^ guided imagery,^57^ and PMR^72^ demonstrated significant improvements in pain post-intervention; one study did not.^69^ One of these 9 studies^68^ reported that a significant pain reduction was sustained at the 6-month follow-up. The most frequently used measurement scales were the VAS and MPQ, employed in 6 studies.^52^^,^^57^^,^^62^^,^^68^^,^^69^^,^^72^

### Fatigue

#### Mind-body therapies versus no control

Out of 3 studies^51^^,^^61^^,^^63^ investigating mind-body therapy without control groups, 1 study (n = 23) on tai chi^61^ demonstrated significant improvements in fatigue directly post-intervention. However, a follow-up assessment 12 weeks after the 28-week intervention found that the improvements were not sustained.^61^ Various measurement tools were used to assess fatigue including daily diaries and the Fibromyalgia Impact Questionnaire (FIQ) Fatigue subscale.

#### Mind-body therapies versus no specific treatment

Among the 8 studies^45–47^^,^^50^^,^^56^^,^^60^^,^^64^^,^^70^ comparing mind-body therapy to no specific treatment, 3 studies (n = 185) on guided imagery, tai chi, and MBSR demonstrated significant improvements in fatigue post-intervention.^47^^,^^56^^,^^70^ Among the 5 remaining studies, 3 reported insignificant reductions in fatigue,^45^^,^^46^^,^^60^ one^50^ reported only within-group differences, and one^64^ relied solely on qualitative comments to assess fatigue symptoms. Various measurement scales were used, including the VAS and FIQ fatigue subscales, Fatigue Symptom Inventory (FSI), and SF-36 Energy/Fatigue subscale.

#### Mind-body therapies versus attention control interventions

Of 7 studies^49^^,^^52^^,^^54^^,^^58^^,^^62^^,^^67^^,^^72^ comparing mind-body therapies to an attention control group, 6 studies (n = 268) on movement therapies, including tai chi and qi gong,^49^^,^^52^^,^^54^^,^^62^ guided imagery,^58^ and PMR^72^ showed significant improvements in fatigue post-intervention. The remaining study found no significant differences in fatigue scores following neurofeedback training.^67^ The most commonly used measurement scales were the FIQ Fatigue and SF-36 Vitality subscales.

### Patient global impression of change

#### Mind-body therapies versus no control

One study^63^ without a control group assessed PGIC over a 24-week yoga intervention, revealing no significant improvement.

#### Mind-body therapies versus no specific treatment

Among 3 studies^45^^,^^46^^,^^59^ comparing mind-body therapy to no specific treatment, only 1 study (n = 113) on MBSR^59^ observed significant improvements in PGIC. In this study, a significant advantage for MBSR was observed both post-intervention and at the 12-month follow-up.^59^ In contrast, 2 studies on yoga^46^ and electromyogram biofeedback^45^ observed no significant improvement in PGIC scores at the end of the intervention.

### Multidimensional function

#### Mind-body therapies versus no control

Out of the 4 studies^44^^,^^51^^,^^61^^,^^63^ investigating mind-body therapy without control groups, 3 studies (n = 73) on neurofeedback,^44^ tai chi,^61^ and qi gong^63^ showed significant improvements in multidimensional function post-intervention. However, the study on tai chi found that improvements were not sustained at the 12-week follow-up assessment after completion of the intervention.^61^ All the studies used total FIQ or Fibromyalgia Impact Questionnaire Revised (FIQR) scores to assess multidimensional function.^44^^,^^51^^,^^61^^,^^63^

#### Mind-body therapies versus no specific treatment

Among the 8 studies^43^^,^^45^^,^^46^^,^^48^^,^^53^^,^^55^^,^^59^^,^^60^^,^^64^^,^^66^ comparing mind-body therapy to no specific treatment, 4 studies (n = 339) on MBSR^43^^,^^59^ and qi gong^53^^,^^60^ demonstrated significant improvements in multidimensional function post-intervention. One of the studies on MBSR reported that the improvements were sustained at the 12-month follow-up.^59^ The remaining 4 studies showed no significant improvements in multidimensional function post-intervention.^45^^,^^46^^,^^48^^,^^55^^,^^66^ Outcome measures used included the FIQR, the Spanish version of FIQ (S-FIQ), and the Quality of Life Profile for the Chronically Ill (PLC).

#### Mind-body therapies versus attention control interventions

Among the 9 studies^49^^,^^52^^,^^54^^,^^58^^,^^62^^,^^67^^,^^68^^,^^69^^,^^71^ comparing mind-body therapy to an attention control group, 6 studies (n = 362) on movement therapies, including tai chi and qi gong,^49^^,^^52^^,^^54^^,^^62^ MAT,^68^ and neurofeedback^71^ observed significant improvements in multidimensional function post-intervention. The study on MAT reported that significant improvements were evident up to 6-months post-intervention.^68^ The most common measurement scales used were the FIQ and variations of the FIQR.

### Sleep disturbance

#### Mind-body therapies versus no control

Two studies^51^^,^^63^ (n = 49) conducted without control groups explored movement therapies, including yoga and qi gong, and demonstrated significant post-intervention improvements in sleep quality and disturbances. Both studies measured this outcome using the Pittsburgh Sleep Quality Index (PSQI).

#### Mind-body therapies versus no specific treatment

Out of 5 studies^46^^,^^47^^,^^50^^,^^53^^,^^70^ comparing mind-body therapy to no specific treatment, 3 studies (n = 222) on qi gong,^53^ guided imagery,^50^ and MBSR^47^ demonstrated significant improvements in sleep disturbance post-intervention. However, the study on MBSR found that improvements were no longer significant when excluding participants lost to follow-up.^47^ A retrospective analysis^64^ was performed on the qualitative data from the original RCT on qi gong^53^ and noted that very few participants expressed improvements in sleep. Various measurement scales were used, including the PSQI, FIQR poor sleep subscale, Stanford Sleep Questionnaire (SSQ), and BPI sleep subscale.

#### Mind-body therapies versus attention control interventions

Among the 7 eligible studies^49^^,^^52^^,^^54^^,^^58^^,^^62^^,^^68^^,^^71^ comparing mind-body therapies to an attention control group, 6 studies (n = 403) on movement therapies, including tai chi and qi gong,^49^^,^^54^^,^^62^ guided imagery,^58^ MAT,^68^ and neurofeedback^71^ demonstrated significant improvements in sleep disturbance post-intervention; one did not.^52^ The study on MAT reported that significant improvements were evident up to 24 weeks post-intervention.^68^ The most commonly used measurement scale was the PSQI.

### Depression

#### Mind-body therapies versus no control

Two studies (n = 60) conducted without control groups investigated the effects of tai chi^61^ and neurofeedback^44^ on depression and observed significant improvements post-intervention. In one study,^61^ it was noted that the positive effects on depression scores did not persist following a 12-week period without tai chi practice, indicating a decline in benefits during detraining. The assessment tools used were the VAS (depression), the depression subscale of the Hospital Anxiety and Depression Scale (HADS), and the General Health Questionnaire (GHQ-28).

#### Mind-body therapies versus no specific treatment

Among 6 studies^43^^,^^45^^,^^46^^,^^55^^,^^56^^,^^59^ comparing mind-body therapy to no specific treatment, 3 studies (n = 243) on guided imagery^56^ and MBSR^43^^,^^59^ showed significant improvements in depression post-intervention. The study on guided imagery^56^ only demonstrated a significant reduction when the intervention duration was at least 10 weeks. One study on MBSR^59^ noted significantly greater improvements in depression among individuals practicing MBSR for 2 or more days per week. The remaining 3 studies^45^^,^^46^^,^^55^ reported insignificant changes in depression post-intervention. The most common measurement tool was the depression subscale of the HADS, used by 3 studies.^43^^,^^55^^,^^59^

#### Mind-body therapies versus attention control interventions

Out of 4 studies^49^^,^^54^^,^^57^^,^^62^ comparing mind-body therapy to an attention control group, 3 studies (n = 173) on movement therapies, including tai chi and qi gong,^49^^,^^62^ and guided imagery^57^ reported significant improvements in depression post-intervention; one did not.^54^ The study on guided imagery^57^ observed significant improvements using the Beck Depression Inventory (BDI), but not using the VAS (depression). The most frequently used measurement scale was the depression subscale of the HADS, used by 2 studies.^54^^,^^62^

### Anxiety

#### Mind-body therapies versus no control

Out of the 3 studies^44^^,^^51^^,^^61^ investigating mind-body therapy without control groups, 2 studies (n = 60) on tai chi^61^ and neurofeedback^44^ demonstrated significant improvements in anxiety post-intervention; one did not.^51^ The study on tai chi^61^ reported that this positive effect was not sustained following a 12-week period after the intervention, indicating a decline in benefits during detraining.

#### Mind-body therapies versus no specific treatment

Among the 3 studies^43^^,^^46^^,^^55^ comparing mind-body therapy to no specific treatment, none reported a significant improvement in anxiety post-intervention. These studies involved MBSR^43^^,^^55^ and yoga.^46^ The measurement tools used included the anxiety subscale of the HADS and the FIQR anxiety subscale.

#### Mind-body therapies versus attention control interventions

Out of the 5 studies^49^^,^^54^^,^^58^^,^^62^^,^^67^ comparing mind-body therapy to an attention control group, 4 studies (n = 217) on movement therapies, including tai chi and qi gong,^49^^,^^54^^,^^62^ and guided imagery^58^ demonstrated significant improvements in anxiety post-intervention; one did not. The study on guided imagery^58^ observed significant improvements in trait anxiety, but not in state anxiety. The most frequently used measurement scales were the anxiety subscale of the HADS and the State-Trait Anxiety Inventory (STAI), used by 4 studies.^54^^,^^58^^,^^62^^,^^67^

### Adverse events

#### Mind-body therapies versus no control

Of the 5 studies investigating mind-body therapy without control groups, 3 studies^61^^,^^63^^,^^65^ explicitly reported on AEs, whether they occurred or not. Of these, 1 study on qi gong^63^ observed 10 AEs, including pain (n = 5), headache (n = 1), cooler body (n = 1), discolored hands and feet (n = 1), increased stress (n = 1), and intermittent cough (n = 1).^63^ The remaining 2 studies on tai chi^61^^,^^65^ reported no AEs during the interventions, with 1 study^61^ also noting no AEs during the detraining period. The other 2 studies did not provide any information on AEs, making it unclear whether they tracked them.

#### Mind-body therapies versus no specific treatment

Of the 12 studies comparing mind-body therapies to no specific treatment, 3 studies^53^^,^^59^^,^^70^ explicitly reported on AEs (whether they occurred or not). One study on tai chi reported that no AEs occurred.^70^ Another study on qi gong noted two potentially intervention-related AEs: plantar fasciitis (n = 1) and shoulder pain (n = 1), both of which resolved gradually.^53^ A study on MBSR reported that 3 participants experienced adverse symptoms during and/or after the intervention with considerable frequency, including mild fatigue, intense palpitations, tension, dizziness, headaches, loss of sexual desire, weight gain, and somnolence.^59^ Another 5 participants experienced adverse symptoms only after the MBSR intervention, but at a very low frequency and intensity. The remaining 9 studies did not provide any information on AEs, making it unclear whether they tracked them.

#### Mind-body therapies versus attention control interventions

Of the 10 studies comparing mind-body therapies to an attention control group, 4 studies^49^^,^^52^^,^^54^^,^^62^ explicitly reported on AEs (whether they occurred or not) and observed no occurrences of AEs. These mind-body interventions included tai chi and qi gong.^49^^,^^52^^,^^54^^,^^62^ The remaining 6 studies did not provide any information on AEs, making it unclear whether they tracked them.

## Discussion

The objective of this systematic review was to synthesize the existing evidence on the effectiveness and safety of mind-body therapies for FM and to provide recommendations for future research. Our findings highlight the varied effectiveness of these therapies across different patient-important outcomes and the lack of emphasis on safety assessments within the studies reviewed. Although this review focused on adults with FM, the findings may also be relevant to other clinical conditions that share common neurophysiological mechanisms. Similar to FM, tension-type headache, chronic whiplash-associated disorder, and irritable bowel syndrome share a predominant pain mechanism known as nociplastic pain.^73^^,^^74^ This distinctive source of pain arises from altered function in pain-related sensory pathways in the peripheral and central nervous systems, resulting in increased sensitivity.^75^

### Comparison with previous work

#### Pain

Among the eligible studies, pain was the most frequently reported outcome, appearing in 23 out of 27 studies. This likely reflects the fact that both FM experts and patients consider pain to be the most important symptom domain to assess in clinical trials.^39^^,^^76^ All mind-body therapies, except for MBSR, showed significant pain relief in at least 1 study at post-intervention. Movement therapies, including qi gong and tai chi, were particularly noteworthy, with all 5 studies on each therapy reporting significant pain improvement compared to control groups. This underscores the effectiveness of movement therapies and suggests that qi gong and tai chi may have a superior impact on pain management for individuals with FM, as evidenced by their larger relative effect sizes. Existing reviews on movement therapies have demonstrated conflicting findings. A 2012 systematic review and meta-analysis reported no significant pain reduction compared to controls,^77^ while a more recent systematic review from 2015 found that movement therapy was superior to usual care and attention control in reducing pain post-intervention.^18^ There is a need for high-quality studies with larger sample sizes to confirm these findings.

Among relaxation therapies, we only identified 1 study on PMR and none on autogenic training, compared to 5 studies on guided imagery. A previous systematic review on relaxation therapies for FM reported similar findings.^78^ Moreover, within the current review, biofeedback demonstrated a significant reduction in pain compared to control groups in most studies (2 out of 3), aligning with findings from a 2013 systematic review.^79^

#### Fatigue and sleep disturbance

Fatigue was reported in more than half of the eligible studies (16 out of 27). Among the mind-body therapies, tai chi (4 out of 4 studies) and guided imagery (3 out of 3 studies) displayed the most evidence for a reduction in fatigue at the end of treatment, followed by qi gong (3 out of 4 studies) and yoga (1 out of 2 studies). For sleep disturbance, guided imagery (3 out of 3 studies) and qi gong (3 out of 4 studies) showed the most evidence, followed by tai chi (2 out of 3 studies), yoga (1 out of 2 studies), and PMR (1 out of 1 study). Consistent with our findings, a recent meta-analysis revealed that, when compared to usual care, tai chi significantly improves fatigue and sleep quality in people with FM.^80^ The effectiveness of tai chi in improving these outcomes may be attributed to its incorporation of slow and controlled movements, which promote muscle relaxation and enhance overall muscle function.^81^

#### Multidimensional function

Measures related to multidimensional function were included in 21 of the 27 studies. Qi gong showed the most consistent evidence for a positive effect, with all 5 eligible studies demonstrating significant improvements compared to control groups at post-intervention. This aligns with a 2013 meta-analysis, which concluded that there was low-quality evidence from 4 studies suggesting short-term improvements in multidimensional function for qi gong compared to usual care.^82^ Furthermore, all 3 studies in the present review demonstrated that tai chi significantly improved multidimensional function compared to control groups, consistent with a recent meta-analysis that found similar benefits for tai chi.^80^ In contrast, the evidence for biofeedback was mixed, with fewer than half of the studies (1 out of 3) showing significant improvements, in agreement with a previous meta-analysis.^79^

#### Patient global impression of change

PGIC was the least commonly reported outcome, addressed in only 4 out of 27 studies. Among these, 1 study on MBSR demonstrated a significant improvement compared to controls at the end of the intervention.^59^ Similarly, a systematic review and meta-analysis of 22 RCTs on movement and body awareness therapies for FM found only 1 study that assessed PGIC.^83^ This scarcity of data on PGIC highlights the need for more comprehensive assessments to understand the broader impact of mind-body therapies from the patient's perspective.

#### Anxiety and depression

Anxiety and depression were reported in fewer than half of the eligible studies, with 10 out of 27 studies reporting on anxiety and 12 out of 27 on depression. Tai chi demonstrated the most evidence for a positive effect on anxiety and depression, with all 3 studies showing significant improvements post-treatment. This aligns with a prior systematic review and meta-analysis, which found beneficial effects on anxiety and depression across various patient populations.^84^ Other mind-body therapies, including guided imagery, qi gong, and biofeedback, showed promise in improving anxiety and depressive symptoms in FM. However, the limited number of studies reporting on these outcomes necessitates caution in drawing definitive conclusions. The 2 studies on yoga showed no significant changes in anxiety or depression at the end of treatment. Nonetheless, a recent meta-analysis on yoga for people with rheumatic diseases observed a large improvement in depressive symptoms and a moderate improvement in anxiety compared with control groups.^85^ Further research is needed to explore the effects of mind-body therapies on mental health outcomes in FM due to the limited number of studies and methodological issues in existing research.

#### Safety assessments and reporting

Few studies evaluated the safety of mind-body therapies by monitoring the frequency and types of AEs throughout the treatment period. Only 10 out of 27 studies reported that they systematically tracked AEs in all participants, regardless of whether any AEs occurred, and only 3 of these studies documented at least one AE among participants. This reflects a broader trend in the literature, where AEs related to mind-body therapies are often underreported. For example, a meta-analysis found that over two-thirds of trials on yoga did not assess its safety profile.^86^ Some notable AEs reported in the literature include psychotic symptoms and an increased susceptibility to false memories associated with mindfulness,^87^ as well as musculoskeletal injuries linked to yoga.^86^ Increased efforts to systematically monitor and report AEs would enable healthcare providers and users to make more informed decisions about the use of mind-body therapies. Such efforts would also promote standardization within the field of complementary, alternative, and integrative medicine, allowing for more accurate comparisons of the safety profiles of different mind-body therapies.

#### Long-term effects of mind-body therapies

Few studies (4 out of 7) that performed follow-up assessments reported that the improvements in outcomes were sustained after the completion of the intervention. Limited evidence exists to determine the long-term impact of mind‐body therapies for adults with FM. Similarly, a systematic review by Theadom et al.^18^ revealed that only 3 studies comparing mindfulness meditation therapies with usual care obtained follow-up data ranging from 1 to 3 months post-intervention. This gap limits insights into the sustained benefit of mind-body therapies. One potential explanation for this gap is the lack of feasibility in maintaining consistent mind-body therapy practices at home. Future research may benefit from modifying mind-body interventions to promote adherence through simplified protocols and additional guided assistance.

### Limitations of this review

Our systematic review has some limitations. First, we included only research articles published in English, which may have excluded relevant studies on tai chi, qi gong, or yoga published in non-English journals, such as those in Chinese or Korean, or in journals not included in the 5 databases we searched. Second, the review does not include gray literature or unpublished findings. Third, while we used several keywords and terms to search for specific mind-body therapies, we could not incorporate terms for every individual mind-body therapy used in the treatment or management of FM. Fourth, the small number of studies involving certain types of mind-body therapy (eg, autogenic training, MAT, PMR, and yoga) reduces the robustness of our findings. Fifth, the movement therapies included in this review (eg, qi gong, tai chi, and yoga) share the common feature of combining physical body movement with a meditative mental state; however, other similar movement modalities, such as karate, tae kwon do, kung fu, and judo, were excluded due to insufficient literature meeting our criteria. Given the similarities between these martial arts and practices such as qi gong and tai chi, this exclusion may introduce bias. Future research should explore these modalities using rigorous methodologies to better understand their potential benefits for individuals with FM. Lastly, due to the heterogeneity of interventions and the varying assessment scales used to measure our outcomes, we did not conduct a meta-analysis. As more evidence becomes available on specific mind-body therapies for the treatment of FM, future reviews should include meta-analyses to better assess and interpret the therapeutic effectiveness and safety of these therapies.

### Limitations of included studies

Several limitations were identified in the studies included in this review. First, there were few studies that reported on the safety of mind-body interventions, which limits our understanding of the safety and feasibility of these interventions in clinical practice. Future studies should consistently report on the safety of these interventions to improve result interpretation and ensure participant safety. Second, the evidence for outcomes in this review was limited by the methodological quality of the trials, with only 9 of the 22 included RCTs considered to be of high methodological quality (met ≥65% of criteria). Since robust RCTs are essential for providing reliable recommendations, future trials should focus on accurately reporting randomization procedures and allocation concealment, which generally scored lower in our methodological quality assessment. Third, few studies reported whether improvements in outcomes were sustained post-intervention, limiting our understanding of the longer-term effect of mind-body interventions for adults with FM. Future studies should investigate the sustainability of benefits over extended periods and explore the lasting effects once participants complete their participation in mind-body therapy. Fourth, the heterogeneity in study designs and outcomes highlights the need for more well-designed, controlled trials to establish more conclusive evidence and guide clinical recommendations. A wide range of outcomes and measures was reported between studies, making it difficult to pool results and compare findings. Future trials should consider following the OMERACT initiative for standardization,^39^ incorporating a core set of outcome measures to enhance consistency across clinical trials in FM. Lastly, there was a lack of studies that investigated the dose–response relationship of mind-body therapies.

## Conclusions

This systematic review provides a comprehensive overview and appraisal of the existing literature on the effectiveness and safety of mind-body therapies for FM. Our findings suggest that some mind-body therapies may be effective in improving pain, multidimensional function, fatigue, and sleep disturbance in adults with FM; however, the overall quality of the evidence is low. Traditional movement practices, including qi gong and tai chi, showed the most consistent evidence for improvements in these outcomes at the end of treatment, followed by guided imagery. In contrast, the effectiveness of mindfulness, biofeedback, yoga, and autogenic training remains uncertain due to either low-quality evidence or a lack of recent studies investigating these therapies. The findings of our review can inform researchers, patients, and healthcare providers about the current evidence on specific mind-body therapies for FM, including which outcomes are most likely to improve with each therapy. Future research should investigate the safety, long-term effects, and dose–response relationships of mind-body therapies while adhering to a standardized set of patient-important outcomes.

## Data Availability

The data underlying this article are available within the article itself.
